# Murine Genetic Background Overcomes Gut Microbiota Changes to Explain Metabolic Response to High-Fat Diet

**DOI:** 10.3390/nu12020287

**Published:** 2020-01-21

**Authors:** Zahra Safari, Aurélia Bruneau, Magali Monnoye, Mahendra Mariadassou, Catherine Philippe, Kurt Zatloukal, Philippe Gérard

**Affiliations:** 1 Université Paris-Saclay, INRAE, AgroParisTech, Micalis Institute, 78350 Jouy-en-Josas, France; z.safari@ymail.com (Z.S.); aurelia.bruneau@inra.fr (A.B.); magali.monnoye@inra.fr (M.M.); catherine.philippe@inra.fr (C.P.); 2 Institute of Pathology, Medical University of Graz, Graz 8010, Austria; 3 Université Paris-Saclay, INRAE, MaIAGE, 78350 Jouy-en-Josas, France; Mahendra.Mariadassou@inra.fr

**Keywords:** fecal microbiota transplantation (FMT), antibiotic treatment, high-fat diet (HFD), genetic background, metabolic disease, non-alcoholic fatty liver disease (NAFLD)

## Abstract

Interactions of diet, gut microbiota, and host genetics play essential roles in the development of metabolic diseases. A/J and C57BL/6J (C57) are two mouse strains known to display different susceptibilities to metabolic disorders. In this context, we analyzed gut microbiota composition in A/J and C57 mice, and assessed its responses to high-fat diet (HFD) and antibiotic (AB) treatment. We also exchanged the gut microbiota between the two strains following AB treatment to evaluate its impact on the metabolism. We showed that A/J and C57 mice have different microbiome structure and composition at baseline. Moreover, A/J and C57 microbiomes responded differently to HFD and AB treatments. Exchange of the gut microbiota between the two strains was successful as recipients’ microbiota resembled donor-strain microbiota. Seven weeks after inoculation, the differences between recipients persisted and were still closer from the donor-strain microbiota. Despite effective microbiota transplants, the response to HFD was not markedly modified in C57 and A/J mice. Particularly, body weight gain and glucose intolerance in response to HFD remained different in the two mouse strains whatever the changes in microbiome composition. This indicated that genetic background has a much stronger impact on metabolic responses to HFD than gut microbiome composition.

## 1. Introduction

Non-alcoholic fatty liver disease (NAFLD) is the most common disease among chronic liver disorders in developed and developing countries [1]. NAFLD includes a clinicopathologic spectrum of diseases ranging from simple hepatic steatosis to nonalcoholic steatohepatitis (NASH) that may progress to cirrhosis and cirrhosis-related complications, including hepatocellular carcinoma (HCC). It is also strongly correlated with obesity, insulin resistance (IR), and type 2 diabetes mellitus. The prevalence of NAFLD, including NASH, is also rising in parallel with the increased frequency of obesity [2]. In the last decade, the role of the gut microbiome in obesity [3] and in NAFLD [4] has been revealed. There is also evidence that the gut microbiome plays a role as a link between genetic and phenotypic diversity among genetically different mouse strains in response to dietary challenges [5,6]. A high-fat diet (HFD) is generally applied to stimulate obesity and hepatic steatosis in experimental animals. However, results are variable in terms of weight gain, steatosis, inflammation, and fibrosis. Indeed, the rodent strain, the fat amount in the diet, the nature of the fat, and the duration of treatment may affect the obtained results [7,8].

The C57BL/6J (C57) is an especially good model, mirroring human metabolic deregulations that are found in obesity because, with HFD treatment, these mice develop obesity, steatosis, hyperinsulinemia, hyperglycemia, and hypertension, while they remain lean without metabolic imbalances when fed a chow diet [9]. Different mice strains respond differently to HFD [10,11], and compared to C57, A/J mice can be considered resistant to HFD-induced obesity, hepatosteatosis, and inflammation, although their food intake is the same [12,13,14,15,16]. Surwit et al. clearly revealed that the development of obesity and hyperglycemia in A/J and C57 is a complex interplay of genetic background and diet [15]. Their findings evidently showed that handling of fat in inbred mouse strains mainly depends on genetic differences, and that genetic predisposition can be more crucial than caloric intake in driving obesity development in response to HFD. The low propensity of A/J mice to develop obesity has been previously shown by Kondo et al. [17] and Surwit et al. [16], and was associated with the upregulation of some important genes linked to lipid metabolism in the small intestine [17]. On the other hand, C57 mice are considered obesity-prone with low expression of the same genes. Similarly, A/J mice are also resistant, while C57 mice are prone to develop type 2 diabetes features.

In addition to their effect on the host phenotype, HFDs have a remarkable influence on gut microbiota. In fact, a growing number of studies have reported HFD-induced alterations in gut microbiota in the obesity epidemic. HFD feedings have been related to alterations in the gut microbial structure and its decreased diversity [18,19]. There is also evidence showing that the gut microbiota is a signature of the metabolic phenotypes regardless of differences in diet and host genetic background [20]. In recent years, it has been shown that the gut microbiota in C57 mice determines the susceptibility to develop NAFLD [21], and that the gut microbiota contributes to the different susceptibility to fatty liver of the BALB/c and 129S6 mouse strains [22]. In this context, the goal of this study was to find out the contribution of genetics/microbiota on the metabolic response to HFD in susceptible (C57) and resistant (A/J) mouse strains, and the influence of genetics on microbiota alterations. For that purpose, we analyzed the gut microbiota composition in A/J and C57 mice, and assessed its responses to HFD and antibiotic treatments. Finally, we evaluated the possibility of exchanging the gut microbiota between the two strains to assess the contribution of the gut microbiota in the host response to HFD.

## 2. Materials and Methods

### 2.1. Animals

Procedures were performed according to the European Guidelines for the Care and Use of Laboratory Animals and approved by the French Veterinary Authorities (authorization number 78-60). All conventional male A/J and C57 mice were purchased at 6 weeks of age from the Jackson Laboratory (Jackson laboratory via Charles River, Sulzfeld, Germany). Upon arrival, mice were kept under a 12/12 h light/dark cycle for 1 week for acclimatization. Mice were identified by microchips injected under their skin. All mice were given autoclaved water and γ-irradiated (45 kGy) chow diet (R04-40, SAFE, Augy, France). For each experiment, 3 mice per cage were kept. All cages were kept in one room under the same conditions as mentioned above.

After 1 week of acclimatization, 7-week-old A/J and C57 mice were gavaged twice a day with drinking water (placebo) or 0.25 mL of a cocktail of antibiotics containing 1 mg/mL of ampicillin, 1 mg/mL of colistin, and 45 µg/mL of vancomycin for 5 days (set-up in our lab). Then, all groups were treated with gamma-irradiated (25 KGy) HFD (Research Diets D12492, 60% calories from fat) and autoclaved water ad libitum for 7 weeks.

**Figure 1:**
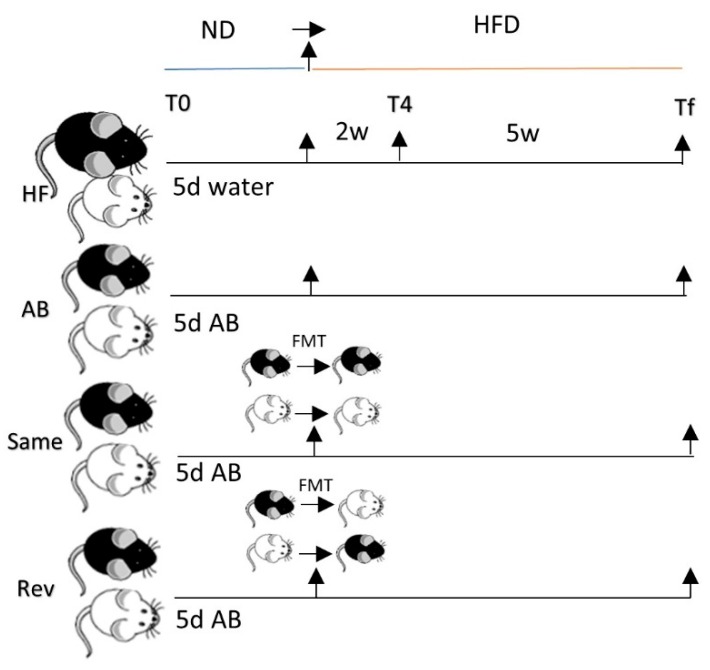
Study design. Study was performed on 4 groups each composed of 12 A/J and 12 C57 BL/6J (C57) mice treated with (1) only HFD; (2) AB and HFD; (3) AB, FMT from same strain, and HFD; (4) AB, FMT from the opposite strain, and HFD. ND, normal diet; HFD, high-fat diet; T0, before HFD treatment; T4, 2 weeks of HFD treatment; Tf, 7 weeks of HFD treatment; D, day; AB, antibiotic treatment; FMT, fecal microbiota transplantation; white mouse, A/J; black mouse, C57.

Our study was composed of 4 groups (Figure 1). Each group was composed of 12 A/J and 12 C57 mice. The first, which corresponded to the control group (HFD), received water by gavage for 5 days to be in the same stress condition as the groups receiving antibiotics. The second group (AB) received antibiotics by gavage, for 5 days. The third group (Same) also received antibiotics by gavage and then a microbiota transplant from a mouse of the same strain (A/J to A/J or C57 to C57). In the fourth group (Rev), microbiota was transplanted from one strain to another following antibiotics treatment (A/J to C57 and vice versa).

Stool pellets were collected from individual mice at 3 time points for microbiota analyses: prior to antibiotic treatment (T0), 2 weeks after HFD treatment (T4), and 7 weeks after HFD treatment (Tf). Fecal samples were also collected after AB treatment to verify bacterial depletion using qPCR analysis. Bacterial DNA concentration was found decreased 1000-fold in the fecal samples of AB-treated mice compared to nontreated mice, indicating nearly complete microbiota depletion.

Donor mice: 1 C57 mouse and 1 A/J mouse were randomly chosen from the initial batch that included all mice from each strain used in this study. They were kept in a separate cage, in the same environment as other mice, with ad libitum chow diet and autoclaved water. On the day of the microbiota transplant, feces were collected from the 2 donor mice. Then, 9 mL sterile water was added to 0.1 g fresh pellet in anaerobic conditions. Each recipient mouse was gavaged once with 0.2 mL of this dilution. In the “Same” group, the C57 mice were gavaged with the diluted feces of the C57 donor, and the A/J mice were gavaged with the diluted feces of the A/J donor. In the “Rev” group, the C57 mice were gavaged with the diluted feces of the A/J donor, and A/J mice were gavaged with the diluted feces of the C57 donor.

Body weight was monitored weekly. All mice were sacrificed immediately after the oral glucose tolerance test. Tissue and plasma samples were collected from the sacrificed mice and stored at −80 °C until analysis.

### 2.2. Oral Glucose Tolerance Test

Oral glucose tolerance tests were performed at the end of the experiment for all mice. Mice were fasted for 8 h before a glucose solution (2 g/kg) was administered by oral gavage. Blood glucose at 0, 15, 30, 60, and 120 min was analyzed from tail-vein blood using an Ascensia Elite XL glucometer (Bayer AG, Zurich, Switzerland). Areas under the glucose curve were calculated following the trapezoidal rule.

### 2.3. Microbial Community Analysis

Extraction of total bacterial DNA from fecal pellets was performed using guanidium thiocyanate and the mechanical bead-beating disruption method as previously described [23]. The V3-4 region of the 16S rRNA genes was amplified using MolTaq (Molzym, Plaisir, France) and primers V3F: TACGGRAGGCAGCAG and V4R: ATCTTACCAGGGTATCTAATCCT. The purified amplicon was sequenced using Miseq sequencing technology (Illumina, Munich, Germany) at the GeT-PLaGe platform (Toulouse, France). Paired-end reads obtained from MiSeq sequencing were analyzed using the Galaxy-supported FROGS (Find, Rapidly, OTUs (Operational Taxonomic Units) with Galaxy Solution) pipeline [24]. For preprocessing, reads with length ≥ 380 bp were kept. Clustering and chimera removal steps followed the FROGS guidelines [24]. Assignation was performed using SILVA 16S. OTUs with abundances lower than 0.005% of the total read set were removed prior to analysis [25]. Then, 16S sequencing data were analyzed using the Phyloseq and ggplot2 R packages in addition to custom scripts. Samples were rarefied to even sampling depths before computing within-samples, also called alpha diversities, (observed richness and Shannon) and between-samples compositional diversities (Bray-Curtis). Principal Coordinate Analysis was also performed on Jaccard dissimilarities to obtain a 2D representation of the samples. Raw, unrarefied OTU counts were used to produce relative abundance graphs and to find taxa with significantly different abundances in A/J and C57 in the control group, or at different time points in the HFD and AB groups. A negative binomial model was fit to each OTU, using DESeq2 with default parameters to estimate abundance log-fold changes. OTUs with low total read counts (<50) or low prevalence (present in less than 25% of the samples, exact threshold specified in each analysis) were filtered out and *p*-values were corrected for multiple testing using the Benjamini-Hochberg procedure to control the False Discovery Rate (FDR).

### 2.4. Statistical Analysis

For metabolic parameters, data are presented as mean values with their standard deviation (SD). Differences were considered significant at *p* < 0.05. Tukey’s multiple comparison tests were used to compare groups with different treatments. Differential abundance of bacteria was tested using negative binomial model implemented in DESeq2 and *p*-values corrected with False Discovery Rate (FDR) procedure. Diversity indices (Chao1 and Shannon) were compared between groups and at different time points using two-way ANOVA. Other statistical analyses of microbiota data were described above. For statistical analysis, R 3.5 software using the package Bioconductor 3.8 and GraphPad Prism 6 were used.

## 3. Results

### 3.1. Metabolic Parameters in C57 and A/J Mice

**Figure 2:**
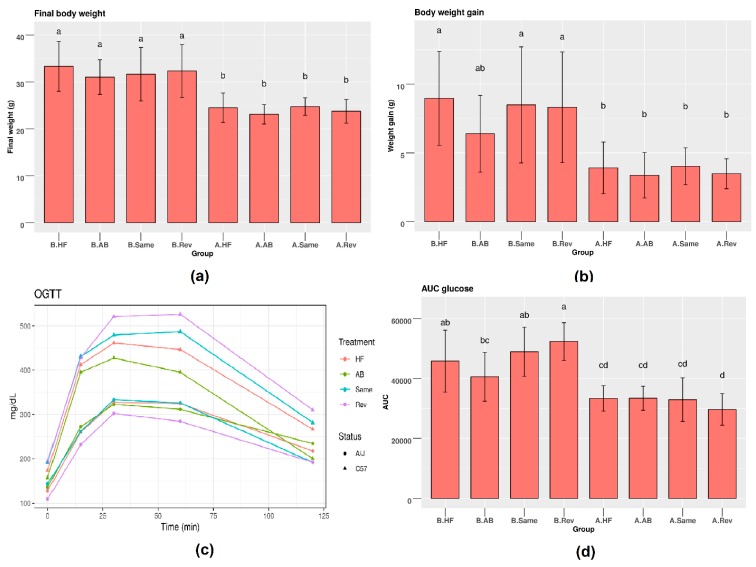
(**a**) Body weight and (**b**) body weight gain in different groups/strains; (**c**) serum glucose levels within 120 min following acute glucose challenge; (**d**) area under the curve (AUC). Mean ± SD, *n* = 12 mice per strain/group. A: A/J, B: C57. Tukey’s multiple comparison tests was used to compare groups with different treatments. *p* < 0.05 was considered significant.

At baseline, no differences were observed in body weight between the different groups of mice. C57 mice showed a significantly higher final weight compared to that ofA/J mice (Figure 2a). Accordingly, HFD feeding increased body weight gain about two-fold more in C57 than in A/J mice (Figure 2b) despite similar food intake. No differences in food intake between the different groups were observed (Supplementary Figure S1), indicating that, in our study, gut microbiota composition did not alter food intake. As expected, weight loss (1 g per mouse) was observed during the antibiotic-treatment week in all AB-treated groups in the two mice strains. AB treatment caused non-significant reduction in the weight gain of C57 mice compared to other treatment groups for this strain, but the final weight gain in A/J mice was not affected by AB treatments. FMT from either C57 or A/J strains increased weight gain in C57 mice compared to their C57-AB counterpart with no transplantation. By contrast, body weight gain in A/J mice was similar in all groups. Comparing the effect of treatment in both strains, only AB treatment was observed to affect body weight gain. Epididymal fat pads were heavier in C57 mice compared to those of A/J in all groups. Similar to body weight, epididymal fat pads tended to have lower weight in the AB group in C57, while no differences were observed between A/J groups. Liver triglyceride concentrations were found higher in C57 compared to A/J mice, but were similar in all groups of each mouse strain (Supplementary Figure S2).

Following HFD, C57 mice showed a higher level of glucose in their serum at different time points compared to A/J mice during the oral glucose tolerance test (OGTT). The area under the curve (AUC) was significantly higher in C57, mice indicating lower glucose tolerance. AB treatment tended to improve glucose tolerance in C57 mice but not in A/J. FMT from the same or opposite strains did not affect glucose tolerance in either strain, indicating that changing the microbiome does not impact differences in glucose tolerance due to the dominating effects of genetics in the two mice strains.

### 3.2. Gut Microbiome of C57 and A/J Mice at Baseline

**Figure 3:**
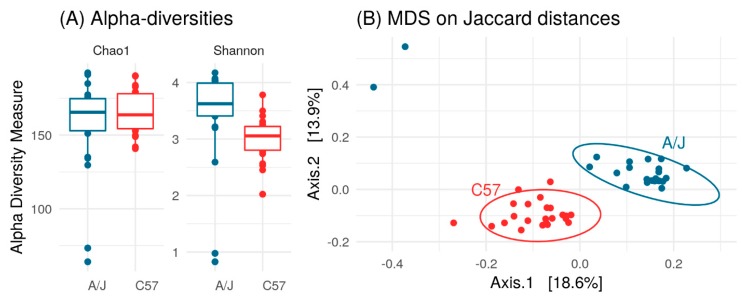
(**A**) Comparison between Shannon and Chao1 diversity indices for C57 (*n* = 23) and A/J (*n* = 23) groups. Chao1 estimates number of species, whereas Shannon estimates effective number of species. A/J and C57 did not differ in terms of Chao1 richness (*p* = 0.28, *t*-test), but A/J had higher Shannon richness (*p* < 0.05, *t*-test). (**B**) Principal Coordinate Analysis (PCoA) plot based on Jaccard distance between samples. A/J and C57 mice exhibited different microbial composition (*p* < 0.001, analysis of similarity test using Adonis function from vegan package with 999 permutations).

With the aim to define if different gut microbiota composition was responsible for the differences in HFD responses between the two mouse strains, we first analyzed the fecal microbiota of mice on a chow diet (T0). The Chao1 diversity index revealed that community richness was the same in C57 and A/J mice indicating a similar number of bacterial species in the two mice strains (Figure 3A). Conversely, the Shannon diversity index was lower in C57 than in A/J mice indicating a biased community structure with fewer dominant bacterial species in C57 (Figure 3A).

Principal-coordinate analysis of the Jaccard distances between the fecal microbiomes exhibited clear differences in the community structure between the two mice strains (Figure 3B). At the phylum level, Bacteroidetes populations were higher in C57 than in A/J mice (Supplementary Figure S3). Accordingly, the proportion of Firmicutes/Bacteroidetes was found higher in A/J than in C57 mice. The *Bacteroidaceae* family was abundant in both strains, but it was higher in C57 mice than in A/J mice. Within the order *Bacteroidales*, Family S24-7 was also observed to be over-represented in the gut microbiome of C57 in comparison to A/J mice. Similarly, the *Alcaligenaceae* family within the *Burkholderiales* order from the Proteobacteria phylum was also found to be more abundant in C57 than in A/J mice. Conversely, the population of *Coriobacteriaceae* was found to be very low in C57 mice, whereas it was one of the more abundant families in A/J mice (Supplementary Figure S4).

**Figure 4:**
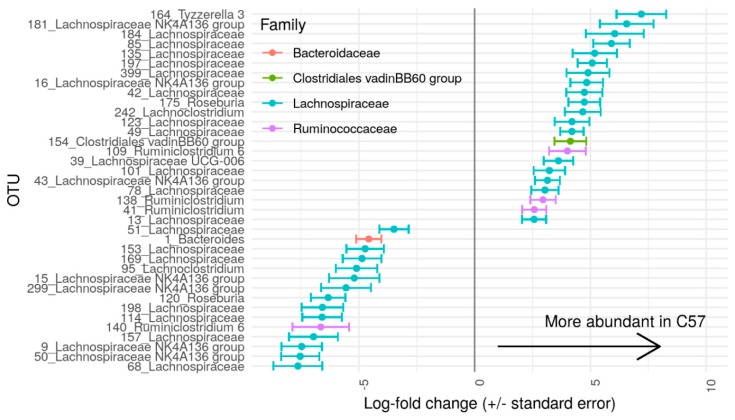
Log fold change of OTUs (Operational Taxonomic Units) with differential abundances (*p* < 0.00011e−4) in A/J (*n* = 23) and C57 (*n* = 23) mice. OTUs present in less than 25% of samples or with read count lower than 50 were filtered out. Differential abundance was tested using negative binomial model implemented in DESeq2 and *p*-values corrected with False Discovery Rate (FDR) procedure.

At baseline, 53 OTUs were significantly different between C57 and A/J mice, namely, 51 Firmicutes, one Bacteroidetes, and one Proteobacteria. At the family level, out of these 53 OTUs, there were 13 *Ruminoccocaceae*, one *Alcaligenaceae*, and one vadinBB60 group from Clostridiales, and the rest were from *Lachnospiraceae* (Figure 4). A total of 27 OTUs were more abundant in C57, and 26 OTUs in A/J mice.

### 3.3. HFD Effect on Microbiome of C57 and A/J Mice

**Figure 5:**
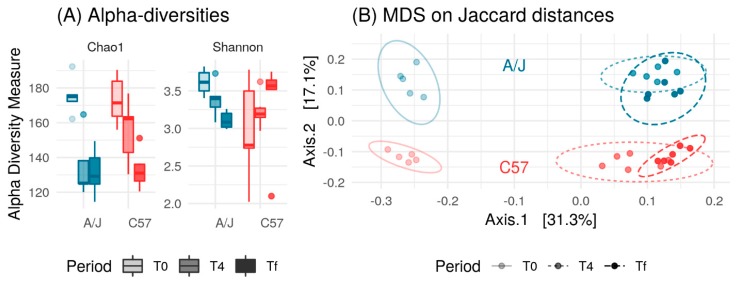
(**A**) Comparison between Shannon and Chao1 diversity indices in A/J (*n* = 6) and C57 (*n* = 6) in response to HFD at different time phases. Time phase had significant impact on Chao1 richness (*p* < 10^-6^, two-way ANOVA) but not Shannon richness (*p* = 0.9, two-way ANOVA). Mice strain had impact neither on Chao1 richness (*p* = 0.23, two-way ANOVA) nor on Shannon richness (*p* = 0.32, two-way ANOVA). (**B**) PCoA plot based on the Jaccard distance between samples. Microbial composition varied with both mouse strain and time phase (*p* < 0.001 for both, analysis of similarity test using the Adonis function from vegan package with 999 permutations).

We analyzed the gut microbiota composition at the three time points in mice from the HFD groups in the two mice strains. It was observed that microbiome richness in both C57 and A/J mice dropped significantly after HFD treatment, which occurred quickly in A/J mice and slowly in C57 mice, leading to a similar level of richness at the end but not the middle of the experiment (Figure 5A). The Shannon diversity index at T0 was higher in A/J compared to that of C57 mice, but decreased remarkably after HFD treatment. In contrast, the Shannon index for C57 mice increased after HFD treatment, suggesting that HFD led to more equal abundances of the bacterial species in C57 mice (Figure 5A).

Based on the evolutionary relationship of the sample’s sequences and their abundance, β-diversity indices (unweighted UniFrac) were used to estimate the distance between samples from both strains in HFD groups, and their shift under HFD treatment during the different time points (T0, before HFD treatment. T4, two weeks of HFD treatment, Tf, seven weeks of HFD treatment; (Figure 5B). Both strains clustered separately at baseline, and their composition markedly shifted along Principal Coordinate Analysis (PCoA) axis 1 in response to HFD. Interestingly, T4 and Tf samples clustered together for each strain, showing that the effect of HFD on microbiota saturated after two weeks (Figure 5B).

**Figure 6:**
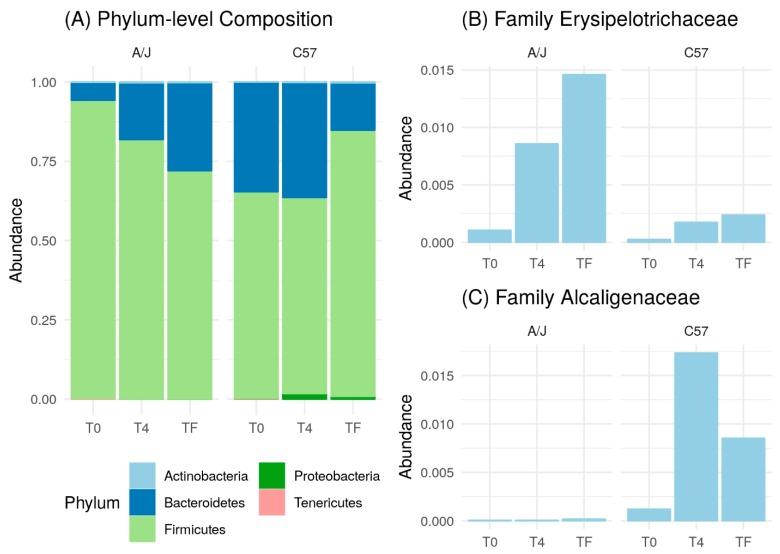
(**A**) Phylum-level composition of A/J (*n* = 6) and C57 (*n* = 6) mice during different time phases; before treatment (T0), two weeks after HFD treatment (T4), after seven weeks of HFD treatment (Tf). Impact of HFD on relative abundances of (**B**) *Erysipelotrichaceae* and (**C**) *Alcaligenaceae* families in A/J and C57 mice.

Then, we compared the effect of HFD on A/J and C57 mice at the phylum level. In C57 mice, Proteobacteria appeared following HFD treatment. Conversely, this phylum was found at significantly lower numbers in A/J mice and did not change markedly under HFD treatment (Figure 6A). At T0, the number of Bacteroidetes in A/J mice was also lower compared to that inC57 mice, while it gradually increased after HFD treatment and reached a higher level at Tf. Conversely, the population of Bacteroidetes in C57 remained similar between T0 and T4 before a marked decrease at Tf (Figure 6A).

We also analyzed the microbiome at the family level. Only families that were found significantly different between the two mice strains are shown. *Erysipelotrichaceae* belonging to the Firmicutes phylum was found more prevalent in A/J than in C57 mice at T0. In both mice strains, HFD triggered an increase in *Erysipelotrichaceae* populations, which was markedly greater in A/J mice (Figure 6B). *Alcaligenaceae* within the Proteobacteria phylum was an abundant bacterium in C57 compared to in A/J at T0. Moreover, the number of *Alcaligenaceae* increased significantly at T4, with a slight reduction at Tf in C57, while its number remained low during the whole HFD treatment in A/J mice (Figure 6C).

**Figure 7:**
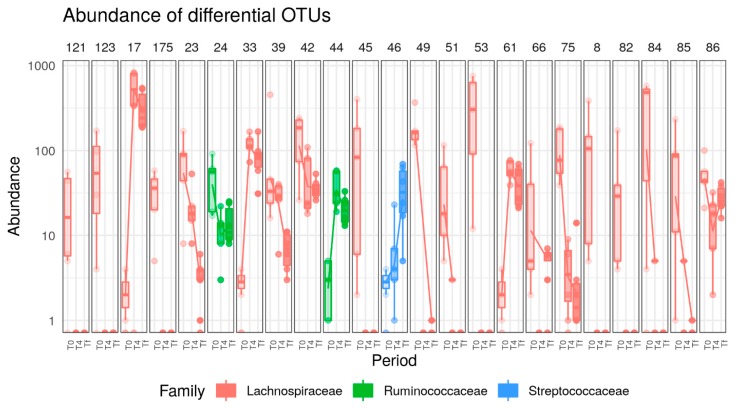
Boxplots of OTUs whose abundance changed (*p* < 0.0001) in response to HFD in A/J mice. OTUs present in fewer than three samples (out of 16) or with read count lower than 50 were filtered out. Differential abundance was tested using negative binomial model implemented in DESeq2 and *p*-values corrected with FDR procedure. Numbers above figure correspond to unique ID of each OTU (e.g., 121 for first one).

In A/J mice, 32 OTUs, all from the Firmicutes phylum were significantly affected by HFD. Under HFD treatment, six OTUs (belonging to the *Blautia*, *Marvinbryantia*, *Lactococcus*, *Tyzzerella*, and *Anaerotruncus* genera) were increased and 26 OTUs (belonging to the *Oscillibacter*, *Lachnoclostridium*, *Lachnospiraceae*, *Lachnospiraceae VCG-006*, *Lachnospiraceae NK4A136,* and *Roseburia* genera) decreased (Figure 7).

**Figure 8:**
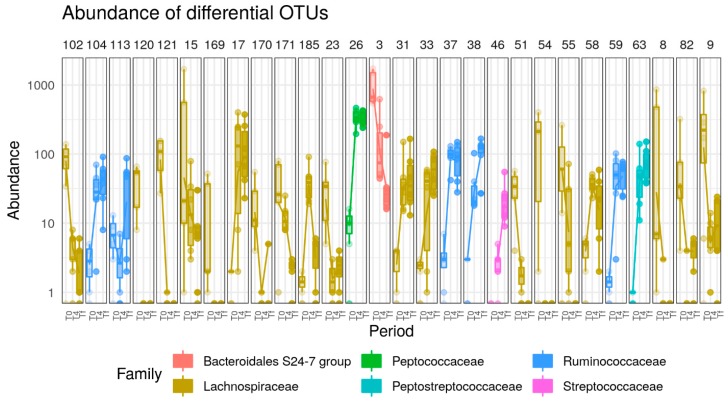
Boxplots of OTUs whose abundance changed (*p* < 0.0001) in response to HFD (*p* < 0.0001) in C57 mice. OTUs present in fewer than three samples (out of 16) or with read count lower than 50 were filtered out. Differential abundance was tested using negative binomial model implemented in DESeq2 and *p*-values corrected with the FDR procedure. Numbers above figure correspond to unique ID of each OTU (e.g., 102 for first one).

In C57 mice, 39 OTUs (37 Firmicutes, one Actinobacteria and one Bacteroidetes) were significantly affected by HFD. Fourteen OTUs increased (belonging to *Blautia*, *Peptostreptococaceae*, *Lactococcus*, *Ruminiclostridium*, *Peptoccocaceae*, *Eubacterium coprostanoligenes* group, *Marvinbryantia*, *Ruminococcaceae*, *Oscillibacter*, *Lachnospiraceae*) and 25 OTUs decreased (belonging to *Lachnospiraceae*, *Lachnospiraceae* NK4A136 group, *Bacteroidales* S24-7 group, *Roseburia*) (Figure 8).

### 3.4. Effect of AB

**Figure 9:**
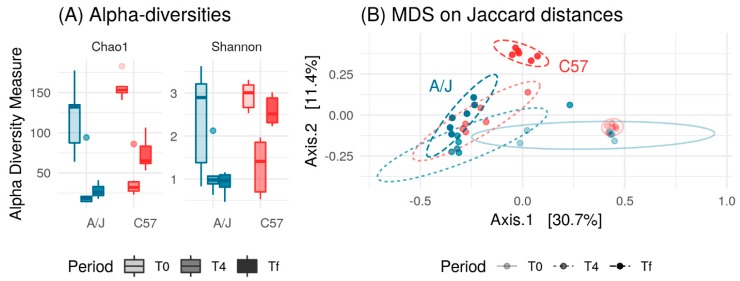
(**A**) Richness (Chao1) and alpha diversity (Shannon) indices in A/J (*n* = 6) and C57 (*n* = 6) microbiota in response to AB treatment. AB treatment had significant impact on both Chao1 richness (*p* < 10^-10^, two-way ANOVA) and Shannon richness (*p* < 0.0001, two-way ANOVAa). Mice strain had an impact on both Chao1 richness (*p* = 0.001, two-way ANOVA) and Shannon richness (*p* = 0.002, two-way ANOVA). (**B**) PCoA plot based one Jaccard distance between samples. Microbial composition varies with both mice strain (*p* = 0.005) and time phase (*p* < 0.001, analysis of similarity test using Adonis function from the vegan package with 999 permutations).

We analyzed the gut microbiota composition at the three time points in mice from the AB groups in the two mice strains. As expected, bacteria richness dropped after AB treatment in both A/J and C57 strains. The Shannon diversity index was reduced in both mice strains after AB treatment, but C57 recovered quickly and returned to close to normal diversity (Figure 9A). Principal-coordinate analysis of the Jaccard distances showed that AB treatment quickly altered the intestinal microbiota of both strains (T4). In accordance to richness and diversity, microbiome profiles in C57 mice continued to change between T4 and Tf, while no further AB effect was noted in A/J mice after T4 (Figure 9B).

A majority of the bacterial clusters were highly decreased by AB treatment and did not recover, particularly in A/J mice. However, specific bacterial species belonging to the *Bacteroides*, *Peptoclostridium*, *Blautia*, and *Enterococcus* genera were increased in both strains following AB treatment, with a more pronounced effect in A/J mice (Supplementary Figure S5). In C57, some of the bacteria diminished by AB recovered at a higher level at the end of the experiment (Tf) (Supplementary Figure S5).

**Figure 10:**
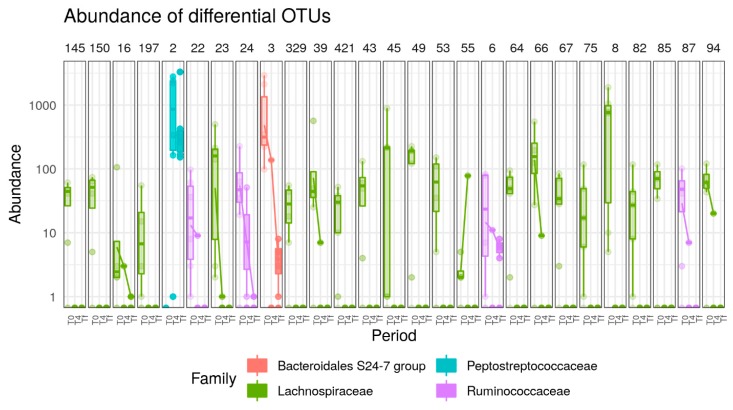
Boxplots of OTUs whose abundance changed (*p* < 0.0001) in response to AB treatment (*p* < 0.0001) in A/J mice. OTUs present in fewer than three samples (out of 16) or with read count lower than 50 were filtered out. Differential abundance was tested using negative binomial model implemented in DESeq2 and *p*-values corrected with the FDR procedure. Numbers above figure correspond to unique ID of each OTU (e.g., 145 for first one). In C57 mice, 23 OTUs (all from Firmicutes phylum) were significantly increased following AB treatment at Tf while one bacterium from *Lachnospiraceae NK4A136* group was decreased (Figure 11).

In A/J mice under AB treatment, 45 OTUs were significantly different between T0 and Tf. Interestingly, they all belonged to Firmicutes, with the exception of one Bacteroidetes OTU, including 26 *Lachnospiraceae*, 16 *Ruminococcaceae*, one *Clostridiales vadinBB60* group, one *Peptostreptococcaceae* and one *Bacteroidales S24-7* group. Only one *Peptostreptococcaceae* was significantly increased at T4 and Tf compared to T0, while all 44 other significantly different OTUs were decreased (Figure 10).

### 3.5. Effect of Microbiome Exchange on C57 and A/J Mice.

Finally, we analyzed the gut microbiota composition at the three time points in mice from the Same and Rev groups in the two mice strains. To compare changes in gut microbiota induced by FMT, we compared the differential OTUs obtained in strains transplanted from the same strain (A/J-Same, C57-Same) and opposite strains (A/J-Rev, C57-Rev). A/J-Same shared more OTUs with C57-Rev than with A/J-Rev, indicating effective transplant of A/J microbiota in C57 mice. Similarly, C57-Same shared more OTUs with A/J-Rev than with C57-Rev, indicating that C57 microbiota was adequately transplanted in A/J mice. However, our results clearly showed that not all bacterial species from one mouse strain were able to colonize the gut of the other mouse strain.

**Figure 12:**
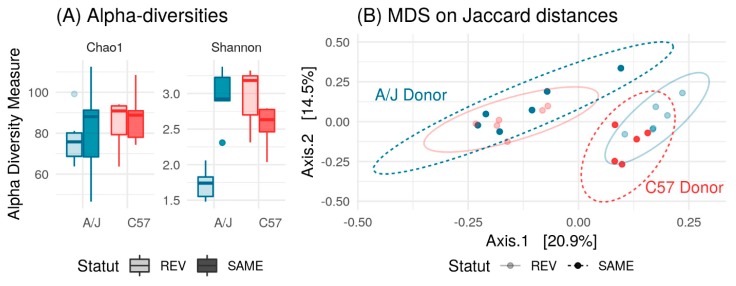
(**A**) Richness (Chao1) and alpha diversity (Shannon) indices in A/J-Rev (*n* = 6), A/J-Same (*n* = 6), C57-Rev (*n* = 6), and C57-Same (*n* = 5) after microbiome transfer groups. Neither mouse strain nor microbiome exchange had significant impact on Chao1 richness (two-way ANOVA with interaction). In contrast, mouse strain, microbiome exchange and their interaction were significant on Shannon richness (*p* < 10^-5^ for all effects, two-way ANOVA with interaction). (**B**) PCoA plot based on Jaccard distance between samples. Microbial composition varied with both mouse strains (*p* = 0.002), status (self vs. exchange) (*p* = 0.012), and interaction (*p* < 0.001, analysis of similarity test using Adonis function from vegan package with 999 permutations).

Richness (Chao1) was not affected by gut microbiota transplants in C57 and A/J mice. Conversely, transplants of C57 microbiota led to a marked decrease in the Shannon index indicating that few bacterial species were dominant in these communities. Coherently, mice associated with A/J microbiota (A/J-Same, C57-Rev) displayed a higher Shannon index (Figure 12A). Remarkably, Principal Coordinate Analysis (PcoA) plots of the microbiota profiles further demonstrated the efficacy of the microbiota exchange. Indeed, A/J-Same and C57-Rev clustered together, as well as C57-Same with A/J Rev (Figure 12B).

**Figure 13:**
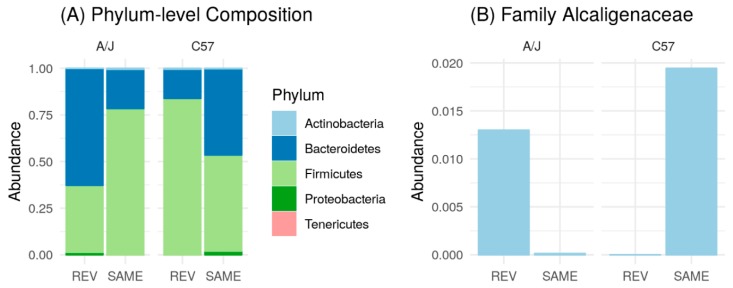
(**A**) Phylum-level composition of A/J-Rev (*n* = 6), A/J-Same (*n* = 6), C57-Rev (*n* = 6), and C57-Same (*n* = 5) groups after microbiome transfer. (**B**) Impact of microbiome exchange on the relative abundance of the *Alcaligenaceae* family.

At the phylum level, the microbiome exchange between the two strains appeared effective. Indeed, C57-Rev and A/J-Rev displayed a microbiota profile close to that of the other strains (A/J-Same and C57-Same, respectively). Mainly, A/J-Rev mice showed a higher prevalence of Bacteroidetes compared to A/J mice receiving a microbiome from an A/J donor (A/J-Same; Figure 13A).

A/J-Rev also harbored Proteobacteria, which were lacking in A/J-Same mice. On the other hand, the C57-Rev group harbored a relatively lower level of Bacteroidetes and higher level of Firmicutes compared to C57-Same (Figure 13A). Consistently, the C57-Rev group showed similar pattern as the A/J-Same group with a relatively lower level of Bacteroidetes and a higher level of Firmicutes compared to C57-Same (Figure 13A). Before microbiota exchange, several bacterial families differed in proportion between C57 and A/J mouse microbiota (Supplementary Figure S4). Most of these families were effectively exchanged between the two mice strains following microbiota transplants. As an example, the *Alcaligenaceae* family was effectively transferred from C57 to A/J strain. Reciprocally, A/J-Same mice, which showed a very low level of *Alcaligenaceae*, could transfer this characteristic to the C57-Rev group (Figure 13B).

## 4. Discussion

The microbiota starts to colonize the gastrointestinal tract just after birth. Then, its composition is impacted throughout life by host genetics and environmental factors including diet, antibiotics, and disease states [26,27]. Numerous studies have demonstrated a role of the gut microbiota in the pathogenesis of many diseases, such as inflammatory bowel diseases, type 2 diabetes, and obesity. How the gut microbiota modifies disease risk in each of these pathologies involves complex interactions with the host’s genetic background [6,28]. Furthermore, studies showed that different mouse strains, but also mice from different suppliers display distinct susceptibilities to obesity and diabetes when challenged with an HFD [28,29] highlighting the role of both genetics and environment in host metabolism. Moreover, the transplant of different microbiota to genetically identical mice can change their phenotype, including steatosis development [21]. Therefore, we can wonder if the gut microbiome of A/J and C57 mice contributes to their different responses to an HFD. To test this hypothesis, four groups of mice from each strain received an HFD for seven weeks. One group (HFD) received no other treatment, while the others received an AB treatment before HFD (AB), an AB treatment followed by a fecal microbiota transplant from the same (Same) or the other mouse strain (Rev).

Under HFD feeding, body weight gain was about twice as high in C57 mice than in A/J mice. This is in accordance with other studies showing higher weight gain after HFD for C57 mice compared to HFD-fed A/J mice [15]. Targeting the gut microbiota using antibiotics or fecal transplants did not affect the final weight of mice in either strain. Whatever the treatment, C57 mice showed lower glucose tolerance compared to A/J mice. In a study performed by Surwit et al. on these two strains after HFD treatment (containing 35.8% fat) for six months, obese C57 mice showed clear-cut diabetes [30]. These results are also in line with the findings of Fraulob et al. on C57 mice fed with 60% high-fat chow for 16 weeks, displaying greater mass gain and impairment of glucose clearance compared to mice fed with standard chow (with 10% fat) [12]. Gallou-Kabani et al. observed that HFD triggered impaired glucose tolerance in both A/J and C57 mice but in contrast with C57 mice, A/J mice remained normoglycemic [13]. In our study, glucose tolerance was significantly different between the A/J and C57 strains in all different treatment groups except for the AB group, indicating that the genetic background of the strains is dominant to environmental changes such as HFD, or microbiome exchange. However, AB treatment slightly improved weight gain and glucose tolerance only in C57 mice, suggesting that the gut microbiota may have a greater influence on this strain’s phenotype. This is also in accordance with the resistance of germ-free C57 mice to diet-induced obesity and IR [31]. Coherently, C57 mice showed reduced inflammation, and improved insulin signaling and glucose metabolism when given antibiotics while obesity-resistant 129S1 and obesity-prone 129S6 mice were not affected by antibiotics treatment [32]. Similarly, Ellekilde et al. suggested that the peak in glucose concentrations in OGTT is mostly affected by the change in gut microbiota in C57 mice [33]. Altogether, these results indicate that HFD driven glucose dysregulations can be improved by antibiotic-induced changes in gut microbiota but that these effects depend on the host’s genetics, with C57 mice being more susceptible to microbiota changes.

In general, the host genetic background is a relatively strong determinant of fecal microbiome composition [34,35]. The two genetically different strains studied here showed different microbiome composition. As initial colonizing microbial species are essential for the developing immune system and establishing an environment in favor of bacterial growth [36], differences in baseline microbiota can help elucidate distinctions in the individual responses to diet [37,38]. We observed differences in the microbiome composition between A/J and C57 strains before any treatment. Particularly, Bacteroidetes was higher in C57 mice and reciprocally, Firmicutes was higher in A/J mice. Furthermore, *Bacteroidaceae*, *Bacteroidales* S24-7 group, and *Alcaligenaceae* were also found to be overrepresented in C57. Conversely, *Coriobacteriaceae* revealed higher abundance in A/J mice. Coherently, Ericsson et al. studied the effects of supplier and genetic background on the composition of the microbiome in C57 and A/J strains, and observed more *Alcaligenaceae* in the C57 mice than in A/J mice (both from the Jax laboratory) at 3.5 and 24 weeks [39]. Interestingly, the Shannon diversity index was found higher in the A/J strain, indicating a more equal abundance of the different bacterial species.

Diet is considered as one of the most critical environmental factors shaping gut microbial structures [40,41,42]. HFD feedings were observed to be linked with alterations in the gut microbial content as well as decreased diversity [19,42,43]. In line with these studies, we found that HFD highly decreased microbiome richness in both strains. Surprisingly, HFD also decreased the Shannon diversity in A/J mice, while it increased it in C57 mice, indicating that the effect of HFD on the microbiome depends on host genetics. Our results also revealed a different effect of HFD on microbiota composition in both mice strains. After HFD treatment, Bacteroidetes decreased in C57 and increased in A/J compared to its initial level. Reciprocally, Firmicutes level increased in C57 and reduced in A/J mice. In addition, Proteobacteria appeared only in the C57 and not in A/J mice after HFD treatment. An increased prevalence of the Proteobacteria phylum was proposed as a dysbiosis marker that could be used as a diagnostic for disease susceptibility [44]. Therefore, we can wonder whether this increase in Proteobacteria contributes to the metabolic disorders observed only in C57 in response to HFD feeding. Gavaging C57 mice with Proteobacteria in order to increase its population could give insight into the contribution of these bacteria to metabolic disorders. The *Erysipelotrichaceae* population was increased by HFD both in C57 and A/J, but the increment was significantly higher in C57 mice. *Erysipelotrichaceae* were associated with dyslipidemic phenotypes, and an increase in *Erysipelotrichaceae* was already observed in mice fed an HFD [45], while four different lineages within *Erysipelotrichaceae* were found to respond differently to diet [46]. Moreover, the abundance of *Erysipelotrichi* was found positively associated with changes in liver fat in humans fed diets with different choline concentrations [47]. *Erysipelotrichaceae* were also linked to lipidemic imbalances in mice and in a hamster model of hypercholesterolemia [46,48]. We speculate that the high number of these bacteria after HFD in C57 mice may cause aggravated metabolic parameters such as higher body weight and glucose intolerance compared to A/J mice. Similarly, *Alcaligenaceae* was increased by HFD only in C57 mice. This bacterium could also be one of the factors aggravating the metabolic phenotype of C57 under HFD treatment, as an increased level of *Alcaligenaceae* in NASH and obese children versus to healthy controls was previously reported by Zhu et al. [49].

As expected, antibiotic treatment significantly reduced Shannon community diversity indices, but the effect was found to be different in the two mice strains. This is in accordance with previous studies showing that modifying microbiota using antibiotics is followed by the re-establishment of the murine intestinal microbiota depending on the genotype of the host [50]. Indeed, diversity remained low eight weeks after antibiotic treatment in A/J while a diverse but different from the initial community, recolonized C57 mice gut. Regarding microbiota composition, *Alcaligenaceae*, which is only seen in C57 mice could survive after AB treatment in this strain. This is in agreement with a study performed by Clarke et al. [51], who found a higher proportion of these bacteria in vancomycin-treated mice compared to lean and Diet-Induced Obesity (DIO) groups. It was also found that antibiotic treatment reduced HFD-induced endotoxemia in mice, along with improvement of the glucose metabolism [52]. Nevertheless, long-term antibiotic administration cannot be considered an appropriate approach for the treatment of obesity and metabolic syndrome [53]. Indeed, low-dose antibiotic treatment in infancy may augment the risk of metabolic dysfunction in adulthood [54]. Similarly, short-term vancomycin treatment in obese humans appears to reduce systemic insulin sensitivity [55]. This is in line with our observations that the interaction between the gut microbiome and glucose level or insulin sensitivity depends on multiple host and environmental factors.

FMT was previously shown to transfer a special phenotype from donor to recipient animal [21,56,57]. For instance, Bäckhed et al. induced for the first time weight gain and increased IR in germfree mice through fecal transplants from their conventional counterparts, despite a simultaneous decrease in food intake [56]. This was attributed to more effective calorie uptake due to carbohydrate processing by the microbiota. It was suggested that an altered gut microbial community, as a primary trigger, is causative rather than consequential by showing the transmissibility of the obese phenotype through fecal transplantation [57,58]. Therefore, exchanging the microbiome between diet-induced metabolic syndrome-susceptible and-resistant strains might change the phenotypes of the recipient mice and give insights into microbiota contribution in mouse physiology. In our study, the microbiome exchange did not markedly affect glucose tolerance in the recipient mice. Moreover, we did not observe a significant change in the weight of the A/J mice receiving a microbiome from C57 mouse and vice versa, compared with mice that received a microbiome from the same strain. This suggests that the genetic effect exceeds the potential influence of the microbiome, and that differences in gut microbiome composition are not responsible for differences in susceptibility to metabolic disorders between A/J and C57 mice. We may also speculate that, despite the success of microbiome transplantation as demonstrated by PCoA, important specific bacterial species from one mouse strain were not able to colonize the other mouse strain. This highlights the importance of recipient genetics on gut microbiota shaping, and may explain why microbiota exchange was not associated with changes in metabolic phenotypes in our study. This is an important point that may have clinical implications as FMT in humans are inevitably performed between subjects who are genetically different. Therefore, the inability of certain bacterial species to colonize the recipient subject may limit the efficacy of FMT if these bacteria are responsible for the beneficial health effects. This suggests that microbiota composition should be deeply analyzed in donor and recipient subjects to identify bacterial species associated with the efficacy or failure of FMT therapeutic effects. Future efforts testing the pathogenic or protective role of individual bacteria revealed here are warranted.

## 5. Conclusions

Here, we described the microbiome diversity and composition of two mice strains with different susceptibilities to metabolic disorders. We found that resistant A/J and susceptible C57 mice harbor distinct microbial communities highlighting the impact of host genetics on microbiota shaping. Moreover, we showed that HFD and antibiotic treatment differently altered the microbiota in the two mice strains, indicating that microbiota structure mainly results from genetic and environmental factors. Following antibiotic treatment, we were able to properly exchange the microbiome composition between the two strains indicating that the majority of the bacterial species from one mouse strain can colonize the other. This did not markedly affect the metabolic phenotype of the recipient mice, suggesting that the gut microbiome does not contribute to differences in metabolic phenotypes between these two mice strains. However, we cannot rule out that the most relevant bacterial species in terms of health effects were not properly transferred from one mouse strain to the other. This incomplete transfer of the gut microbiota should be taken into account when evaluating the efficacy of FMT in humans.

## Figures and Tables

**Figure 11:**
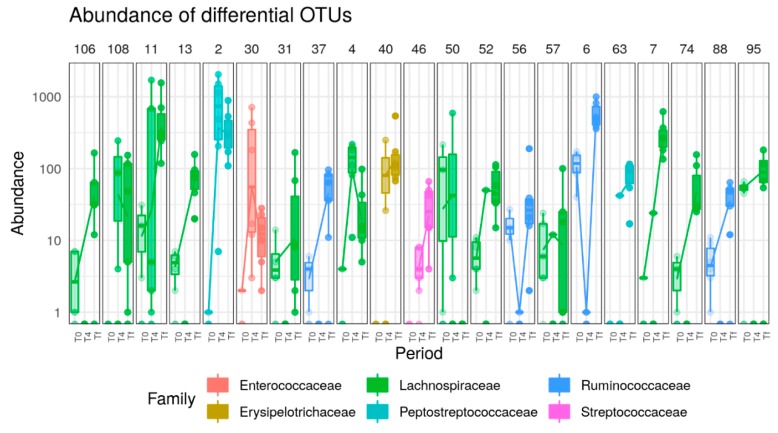
Boxplots of OTUs whose abundance changed (*p* < 0.0001) in response to AB treatment (*p* < 0.0001) in C57 mice. OTUs present in fewer than three samples (out of 16) or with read count lower than 50 were filtered out. Differential abundance was tested using negative binomial model implemented in DESeq2 and *p*-values corrected with the FDR procedure. Numbers above figure correspond to unique ID of each OTU (e.g., 106 for first one).

## Supplementary Materials

The following are available online at https://www.mdpi.com/2072-6643/12/2/287/s1, Figure S1: Food consumption, Figure S2: Metabolic parameters, Figure S3: Phylum differences between A/J and C57 before treatment, Figure S4: Family differences between A/J and C57 before treatment, Figure S5: Genus differences between A/J and C57 mice in different study groups and different time phases.
