# Supplementary material for: Murine Genetic Background Overcomes Gut Microbiota Changes to Explain Metabolic Response to High-Fat Diet
Source: Nutrients. 2020 Jan 21;12(2):287. doi: 10.3390/nu12020287 (PMC7071469; doi:10.3390/nu12020287)
Supplement: Supplementary file 1 [file nutrients-12-00287-s001.zip › Additional files/Food_consumption.pdf]

Average daily food consumption of HF group

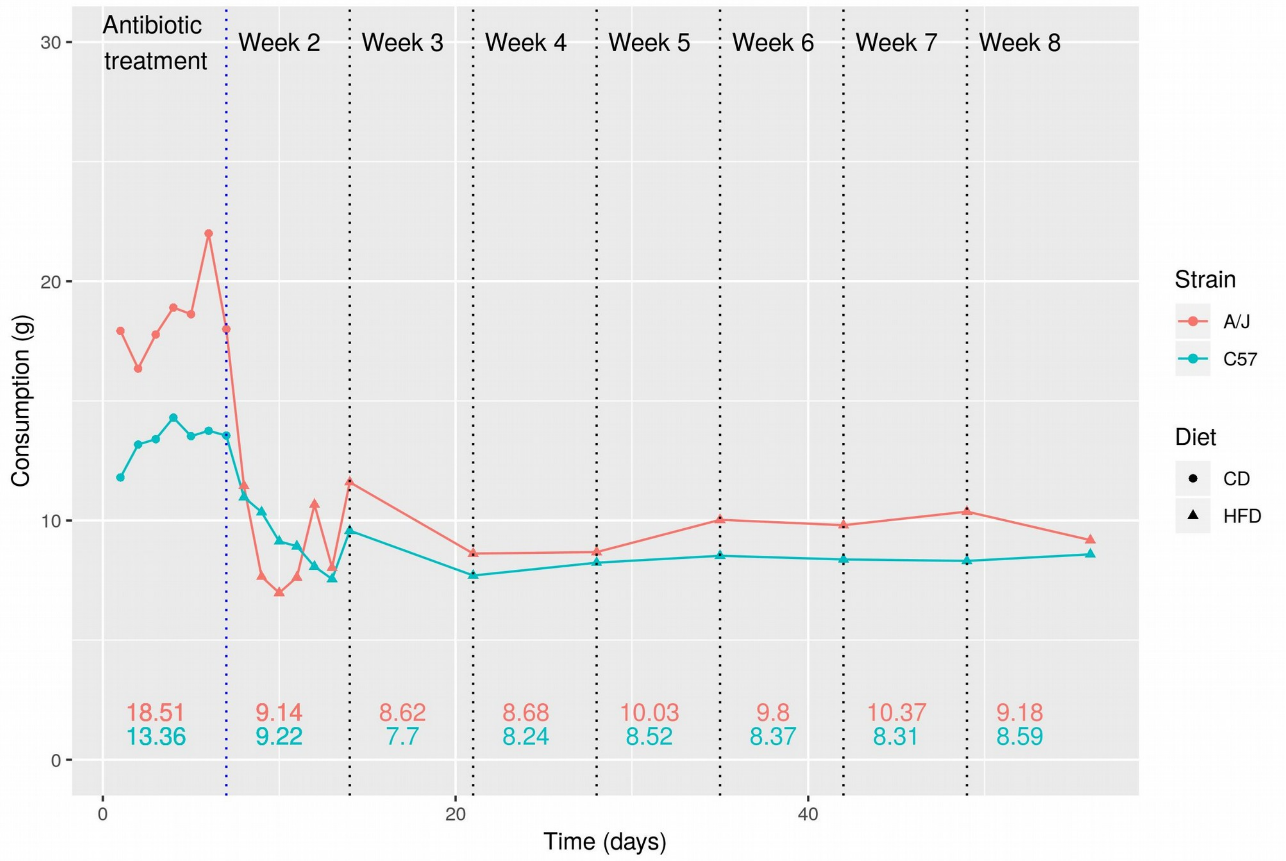

The food consuption by A/J and C57 strain indicated by red and blue colour in the HF group during the time of experiment with the weekly mean of food intake for each strain.

Average daily food consumption of AB group

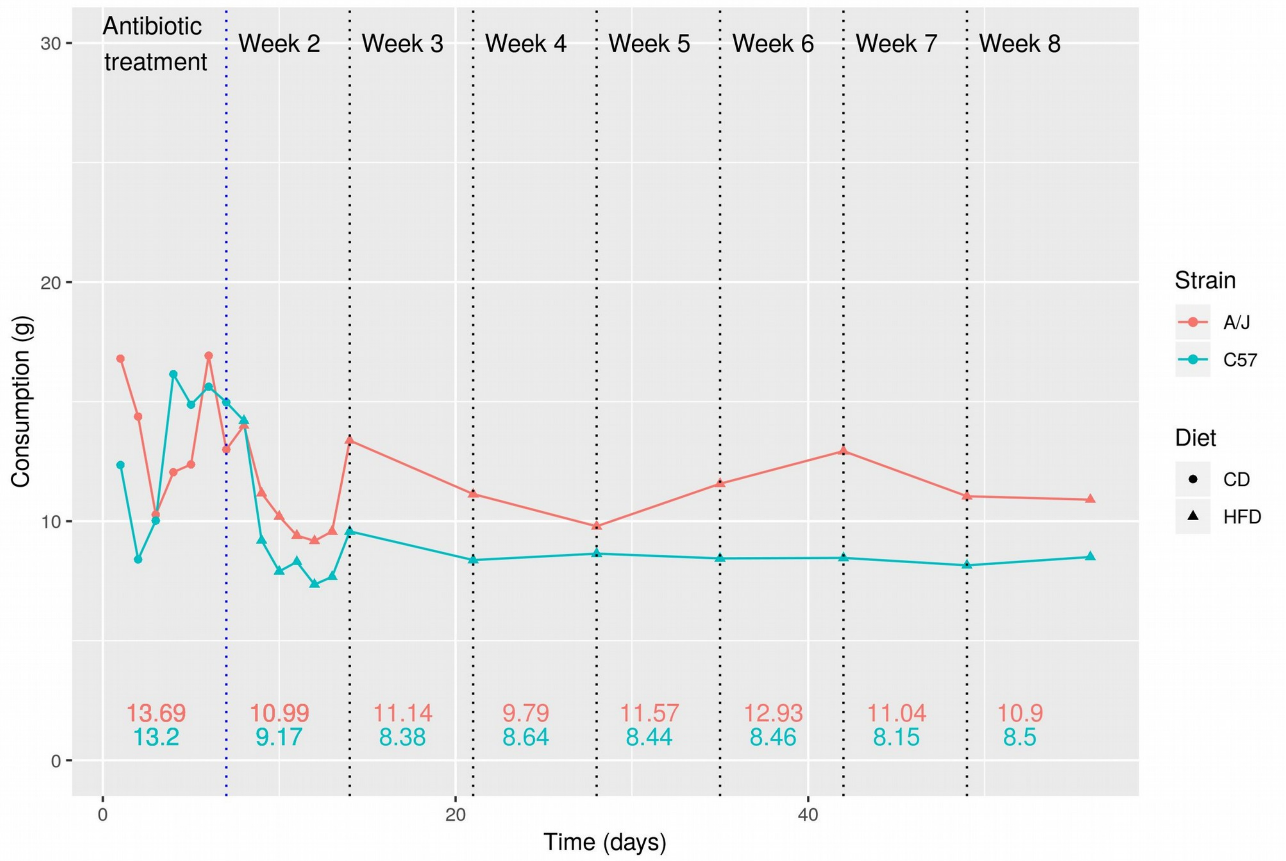

The food consuption by A/J and C57 strain indicated by red and blue colour in the AB group during the time of experiment with the weekly mean of food intake for each strain.

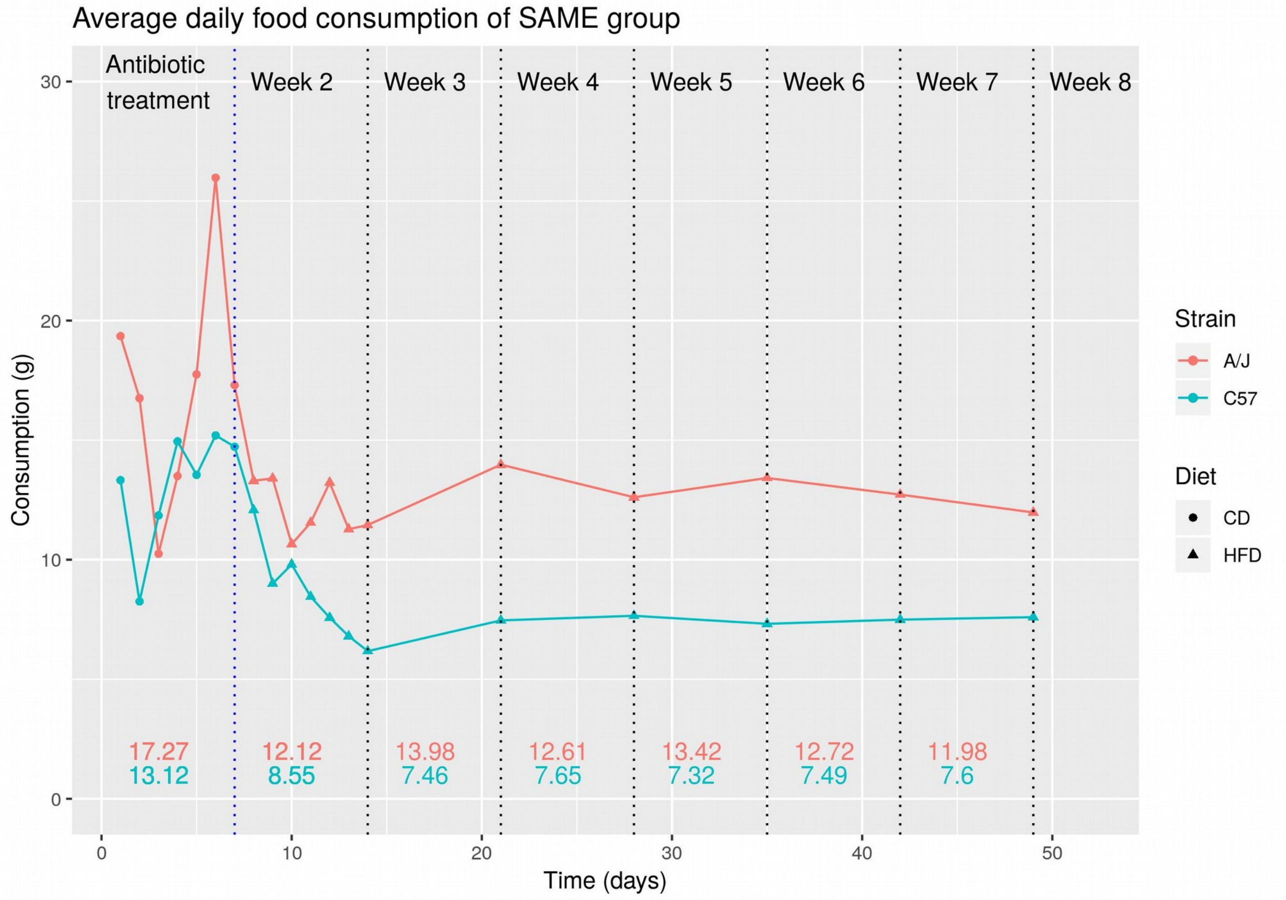

The food consuption by A/J and C57 strain indicated by red and blue colour in the SAME group during the time of experiment with the weekly mean of food intake for each strain.

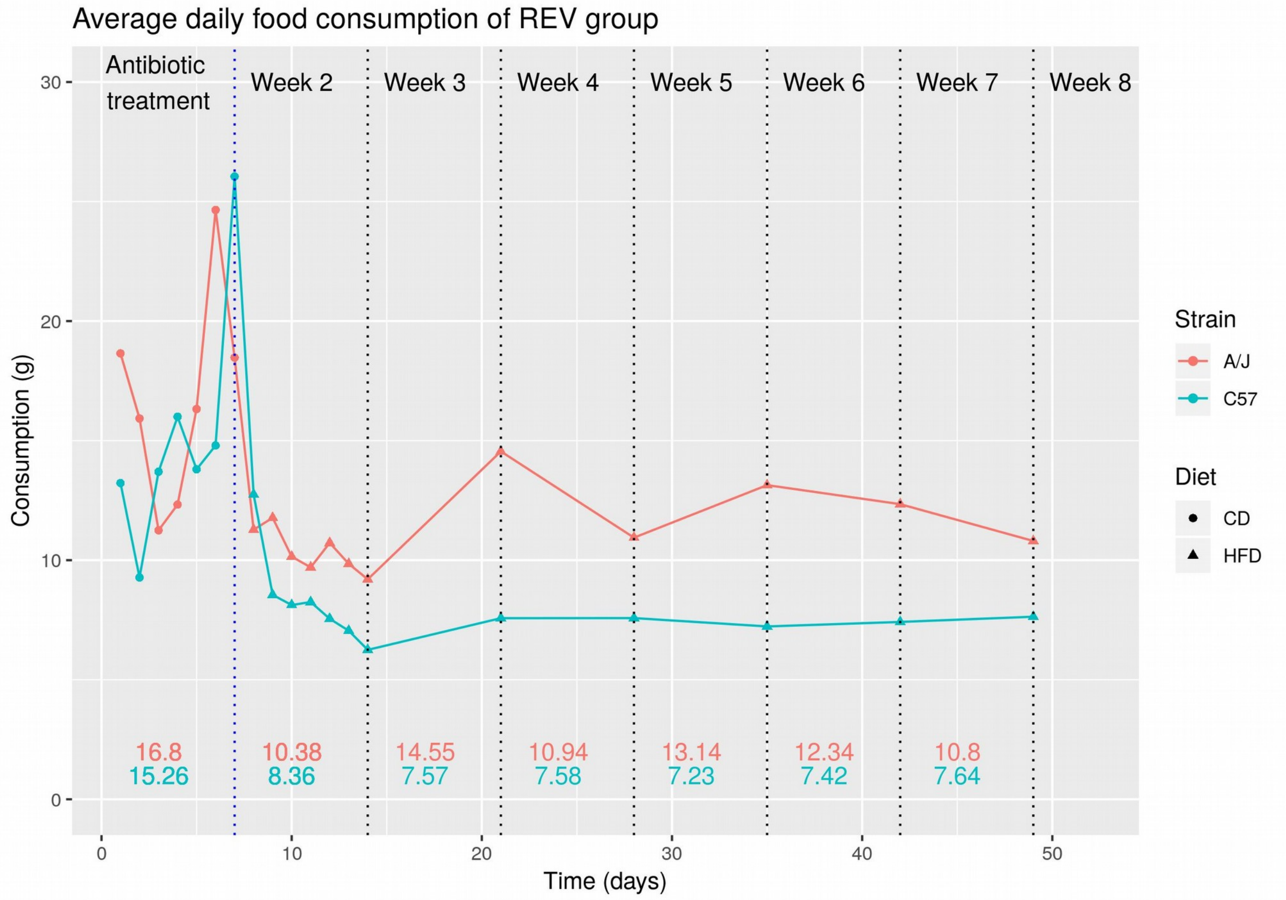

The food consuption by A/J and C57 strain indicated by red and blue colour in the REV group during the time of experiment with the weekly mean of food intake for each strain.
