# Supplementary material for: Murine Genetic Background Overcomes Gut Microbiota Changes to Explain Metabolic Response to High-Fat Diet
Source: Nutrients. 2020 Jan 21;12(2):287. doi: 10.3390/nu12020287 (PMC7071469; doi:10.3390/nu12020287)
Supplement: Supplementary file 1 [file nutrients-12-00287-s001.zip › Additional files/Genus_Difference.pdf]

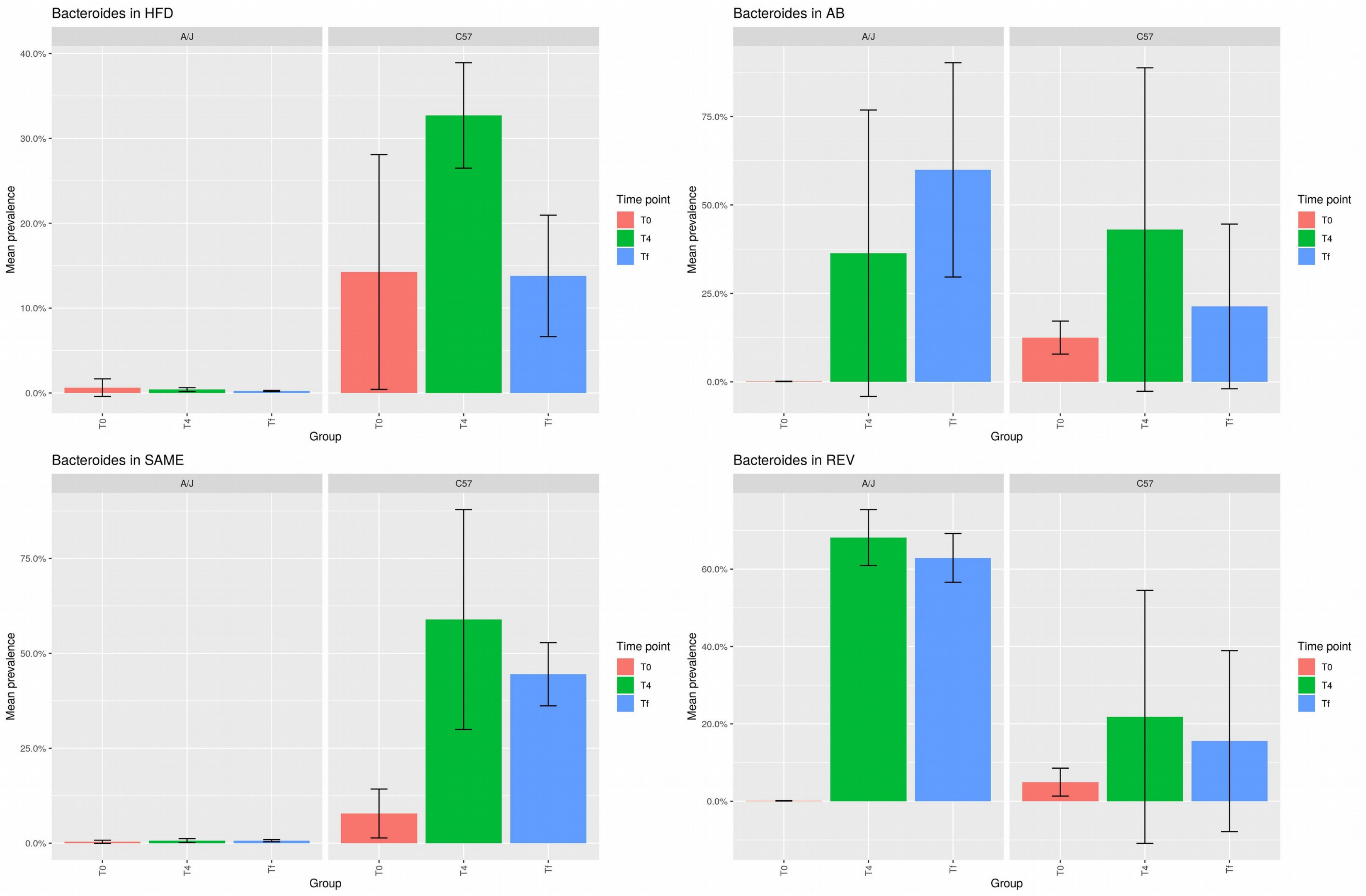

The mean prevalence of Bacteroides in A/J and C57 strain at different time points for different treatment groups.

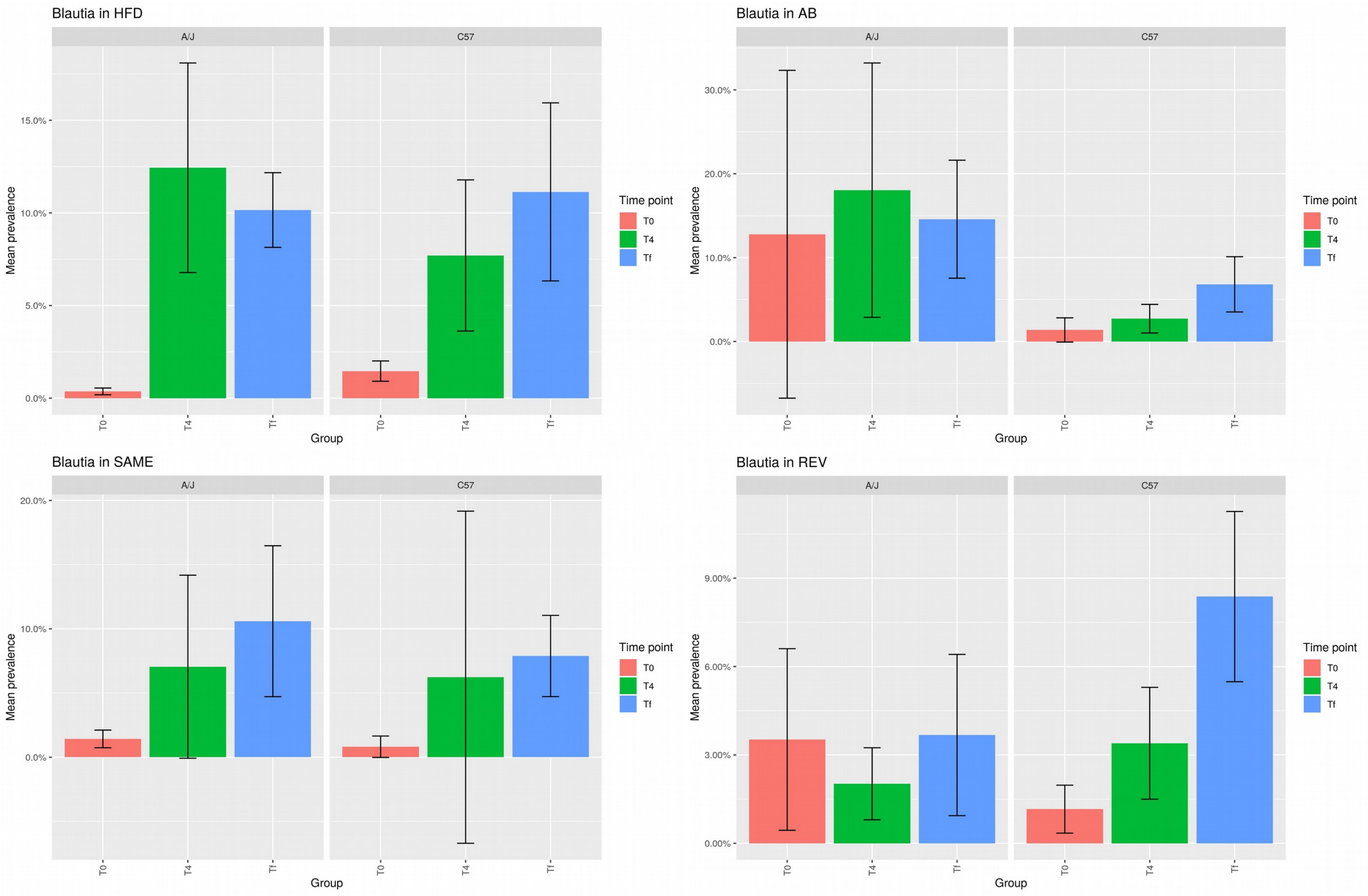

The mean prevalence of Blautia in A/J and C57 strain at different time points for different treatment groups.

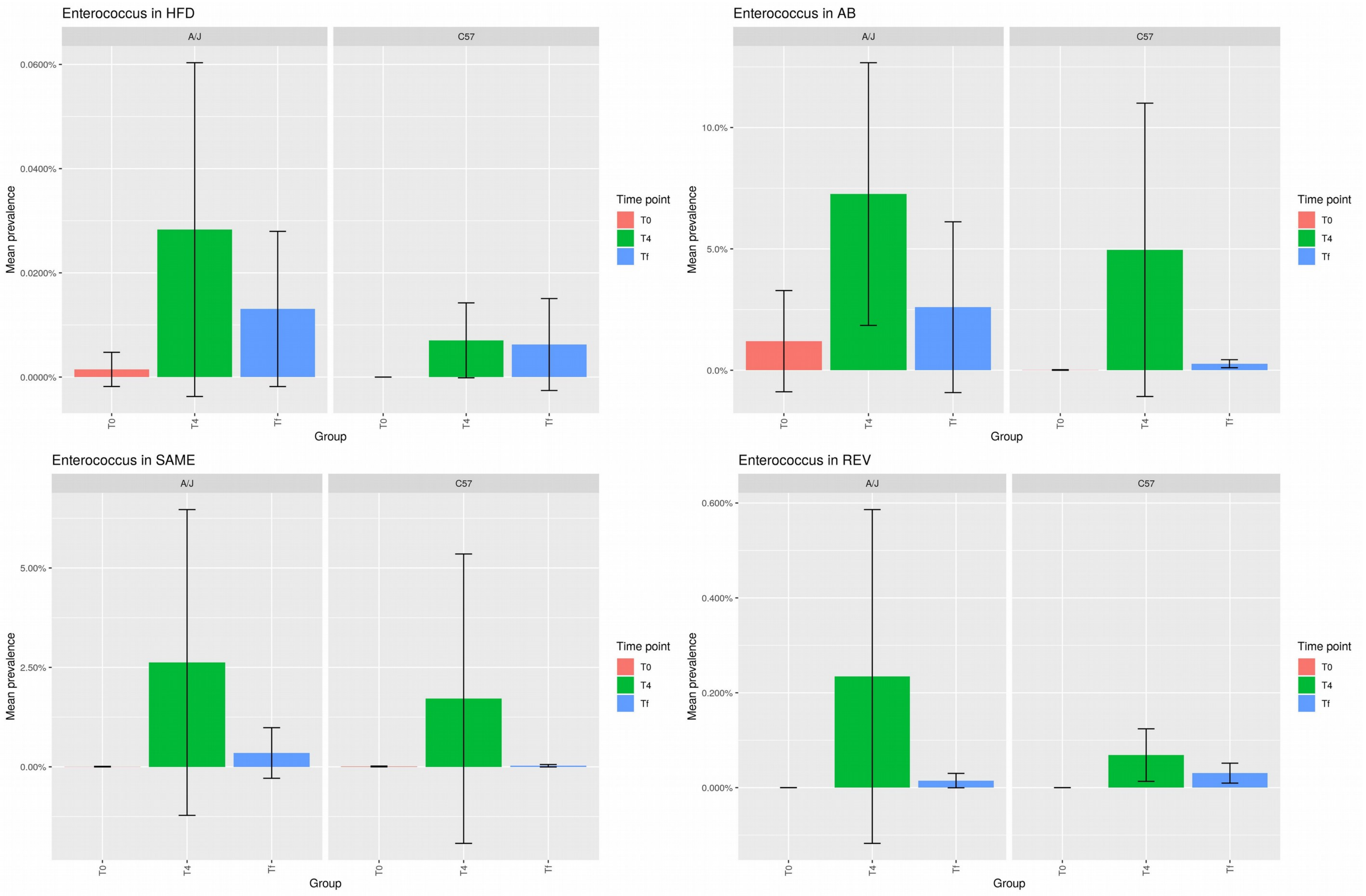

The mean prevalence of Enterococcus in A/J and C57 strain at different time points for different treatment groups.

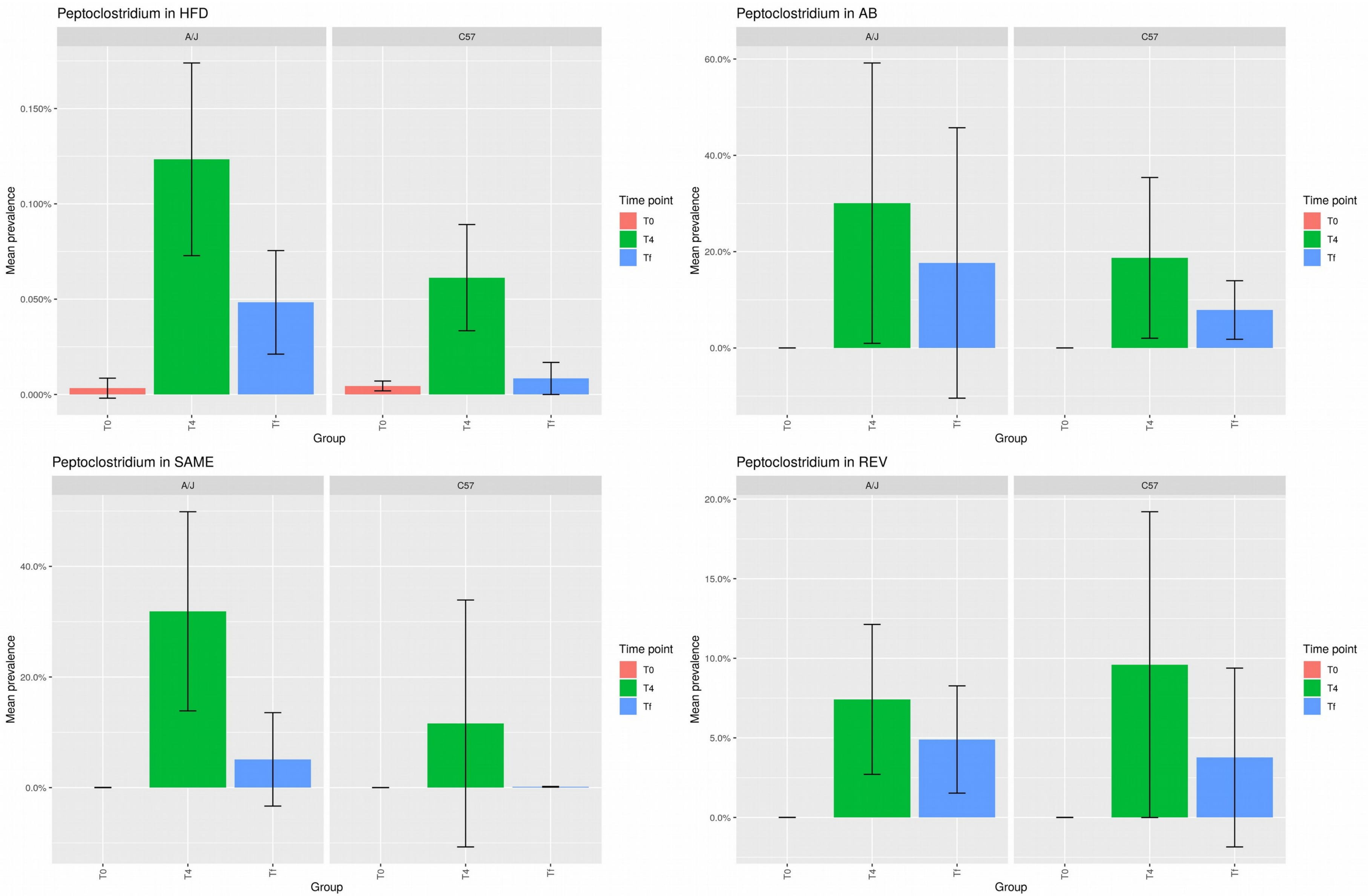

The mean prevalence of Peptoclostridium in A/J and C57 strain at different time points for different treatment groups.
