# Supplementary material for: Murine Genetic Background Overcomes Gut Microbiota Changes to Explain Metabolic Response to High-Fat Diet
Source: Nutrients. 2020 Jan 21;12(2):287. doi: 10.3390/nu12020287 (PMC7071469; doi:10.3390/nu12020287)
Supplement: Supplementary file 1 [file nutrients-12-00287-s001.zip › Additional files/Metabolic_Parameters.pdf]

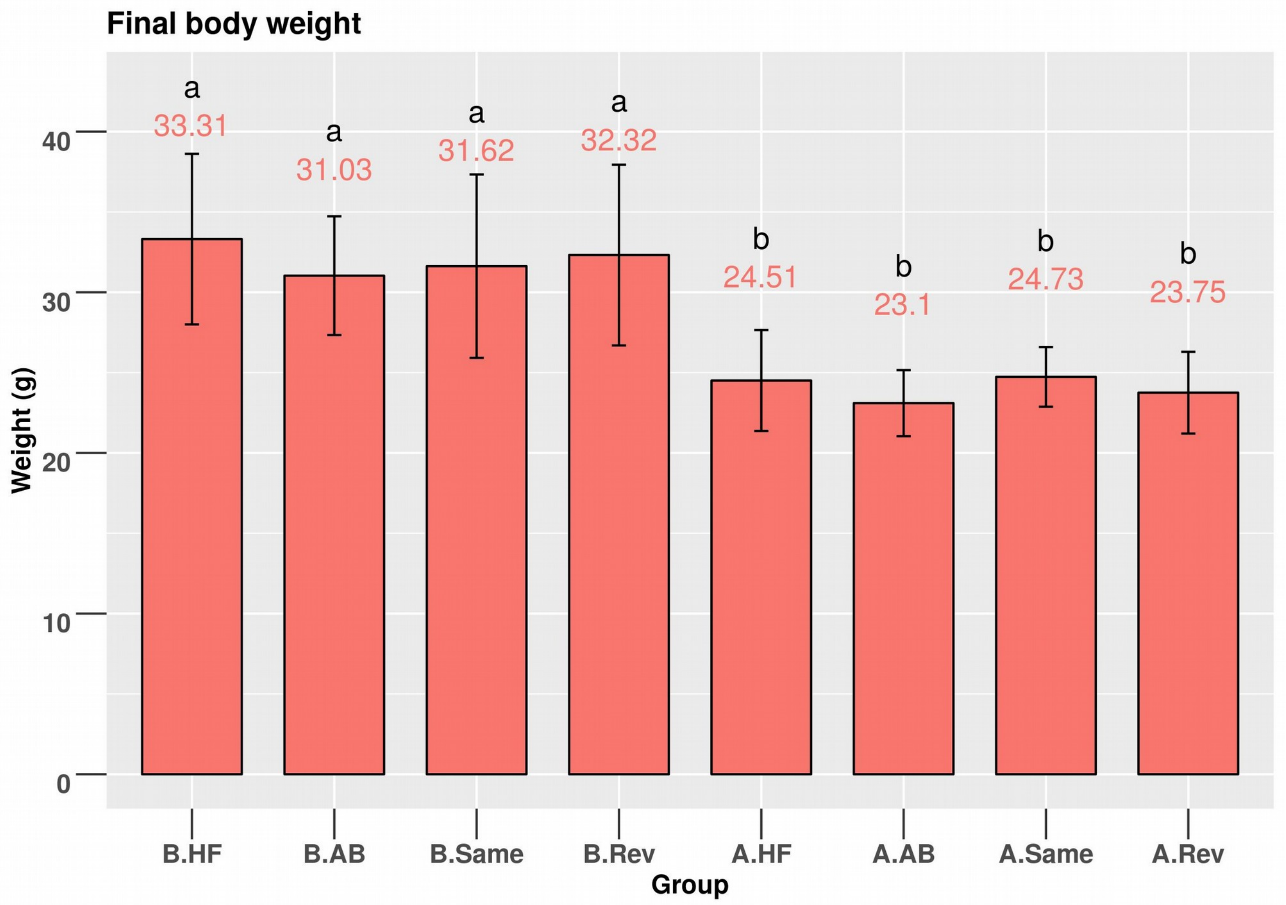

The final body weight of A/J and C57 mice in different experimantal groups. A and B stand for A/J and C57, respectively.

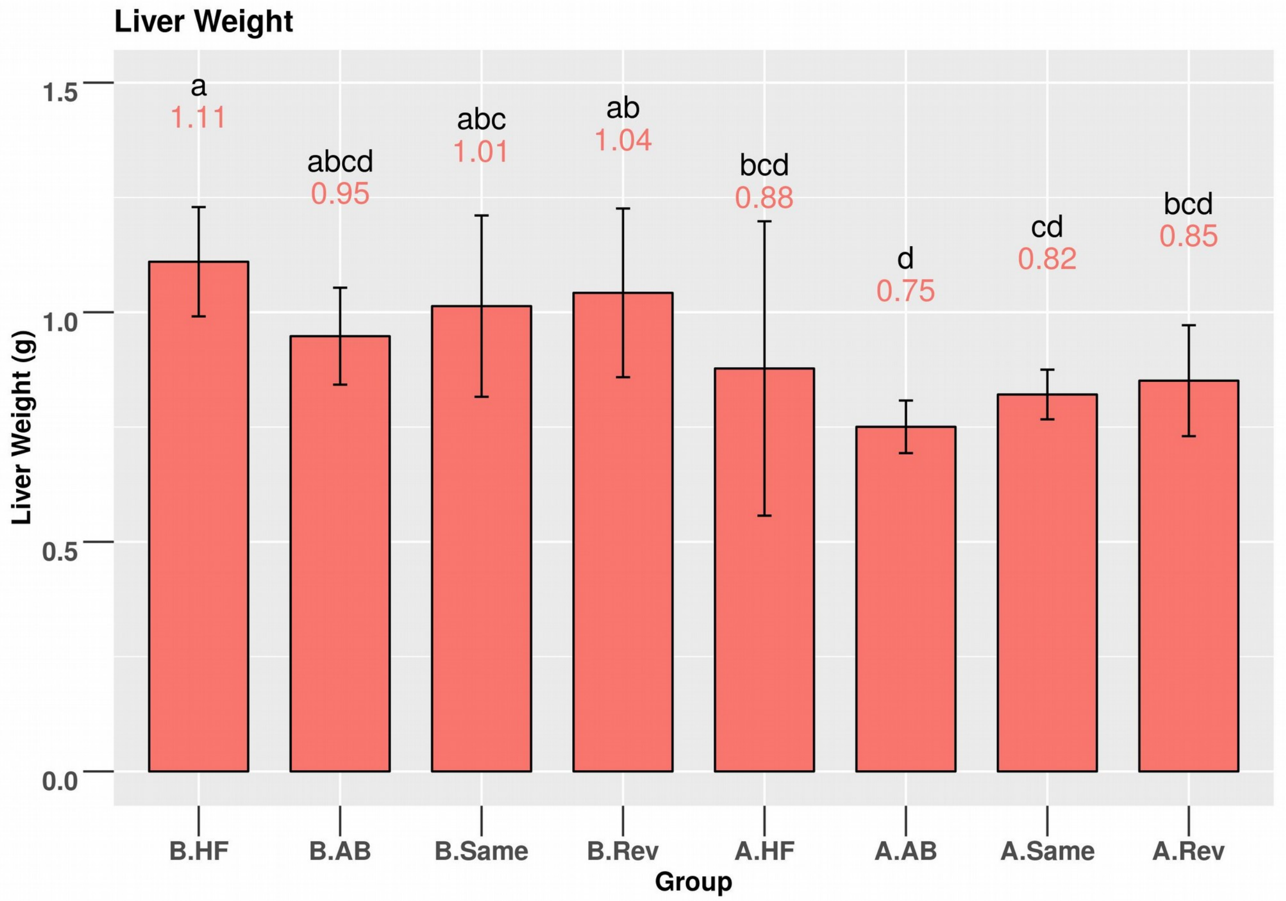

The liver weight of A/J and C57 mice in different experimantal groups. A and B stand for A/J and C57, respectively.

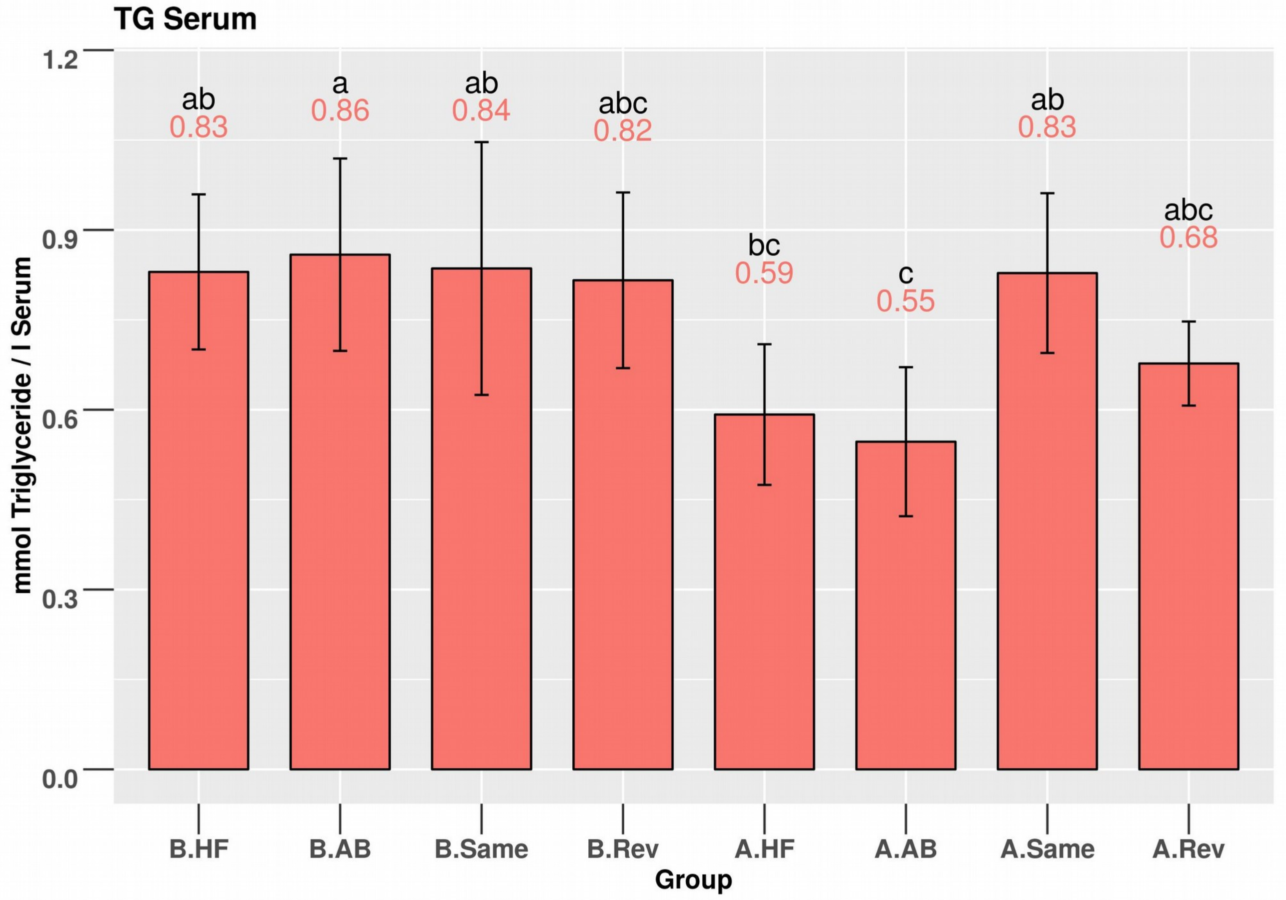

The triglyceride amount in the serum of A/J and C57 mice in different experimantal groups. A and B stand for A/J and C57, respectively.

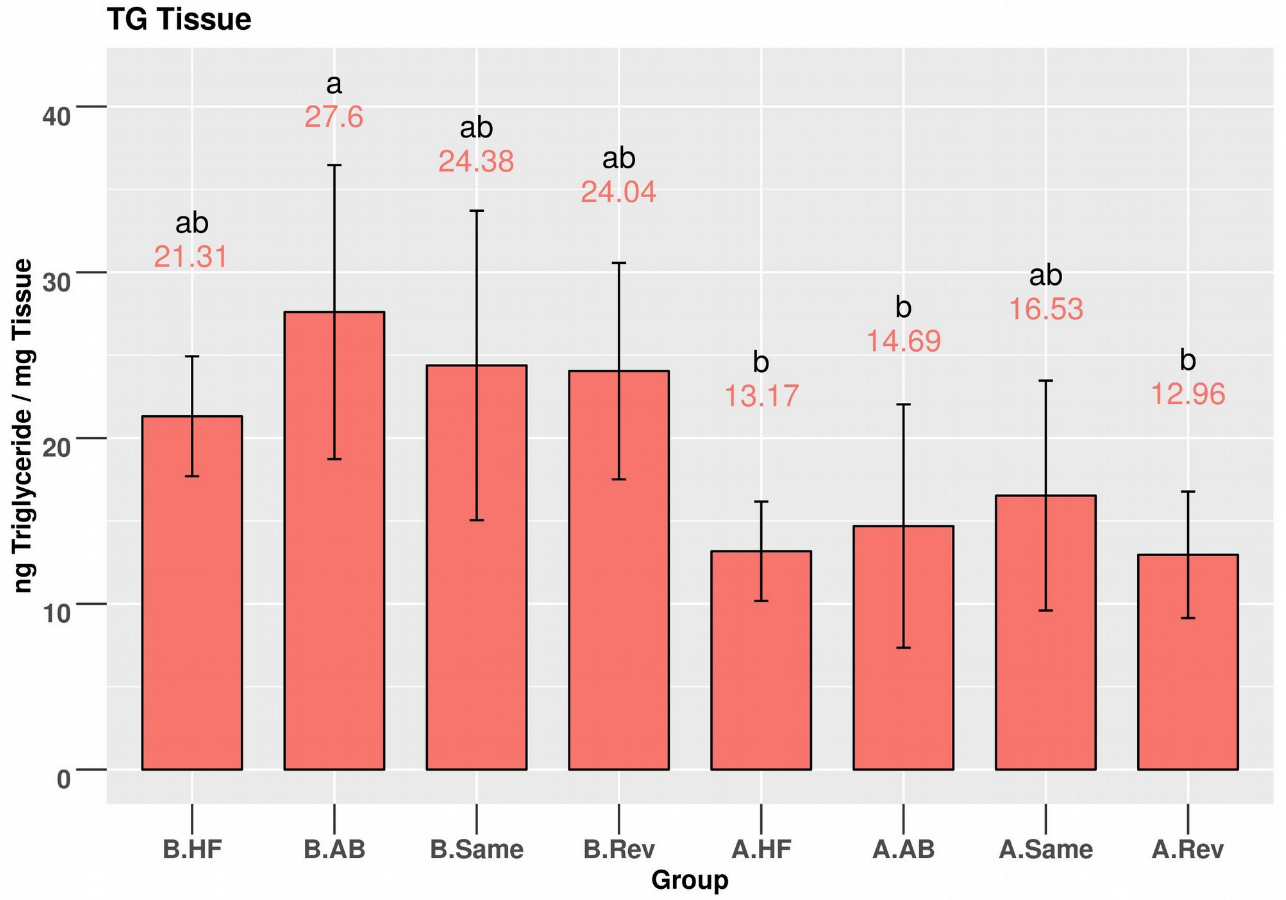

The triglyceride amount in the liver tissue of A/J and C57 mice in different experimantal groups. A and B stand for A/J and C57, respectively.

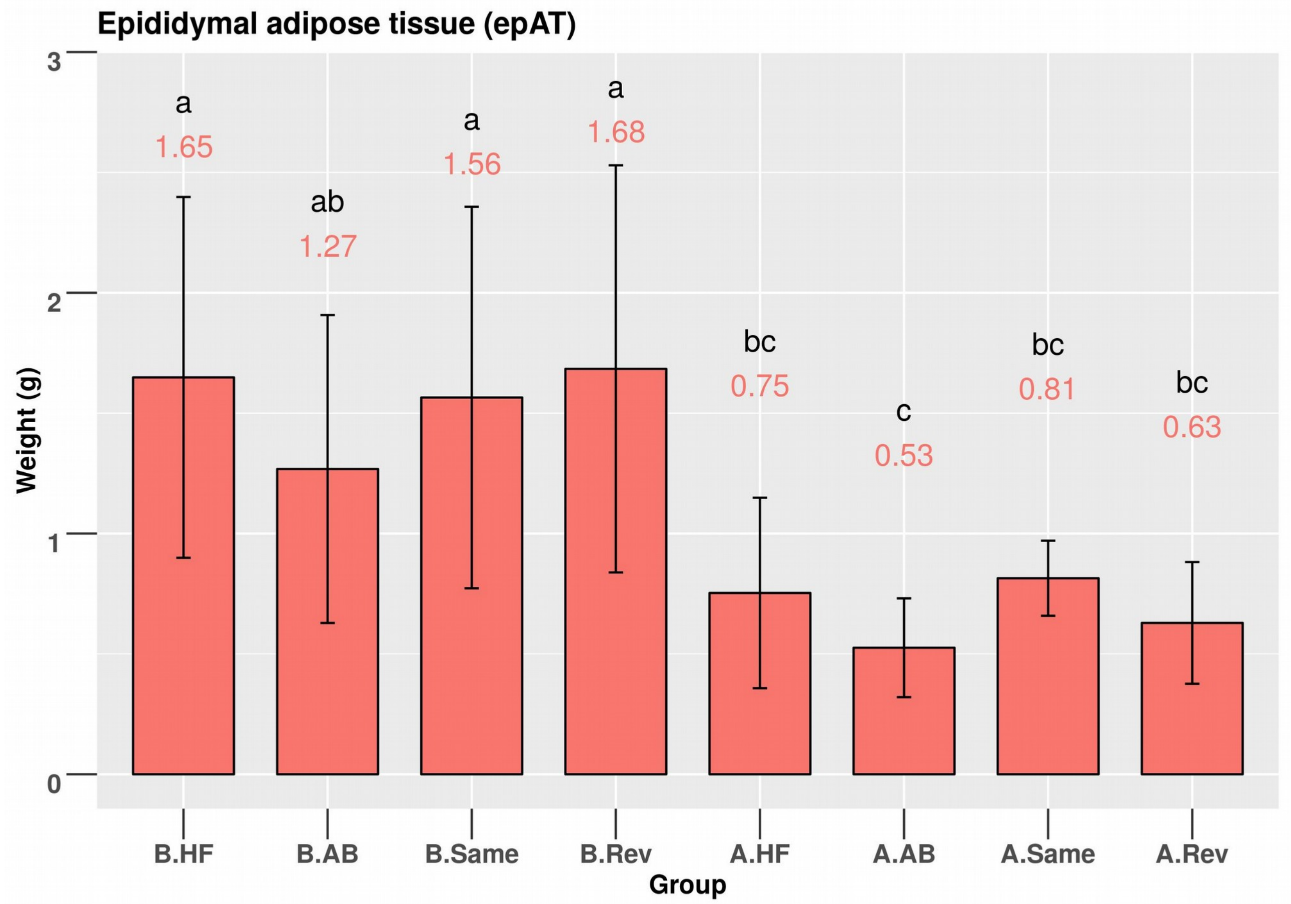

The epididymal adipose tissue weight of A/J and C57 mice in different experimantal groups. A and B stand for A/J and C57, respectively.
