# Supplementary material for: Murine Genetic Background Overcomes Gut Microbiota Changes to Explain Metabolic Response to High-Fat Diet
Source: Nutrients. 2020 Jan 21;12(2):287. doi: 10.3390/nu12020287 (PMC7071469; doi:10.3390/nu12020287)
Supplement: Supplementary file 1 [file nutrients-12-00287-s001.zip › Additional files/Phylum_Difference.pdf]

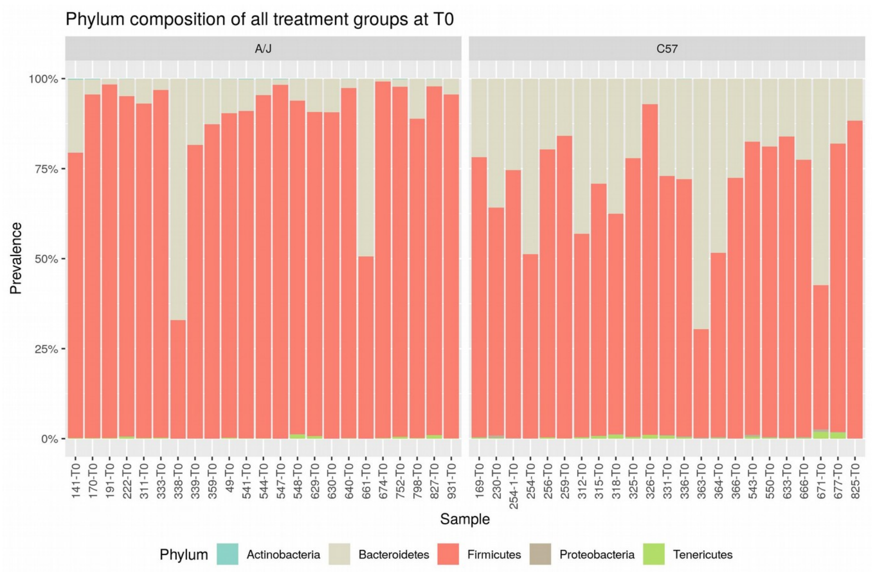

Different microbiome composition in the phylum level between A/J and C57 strain.

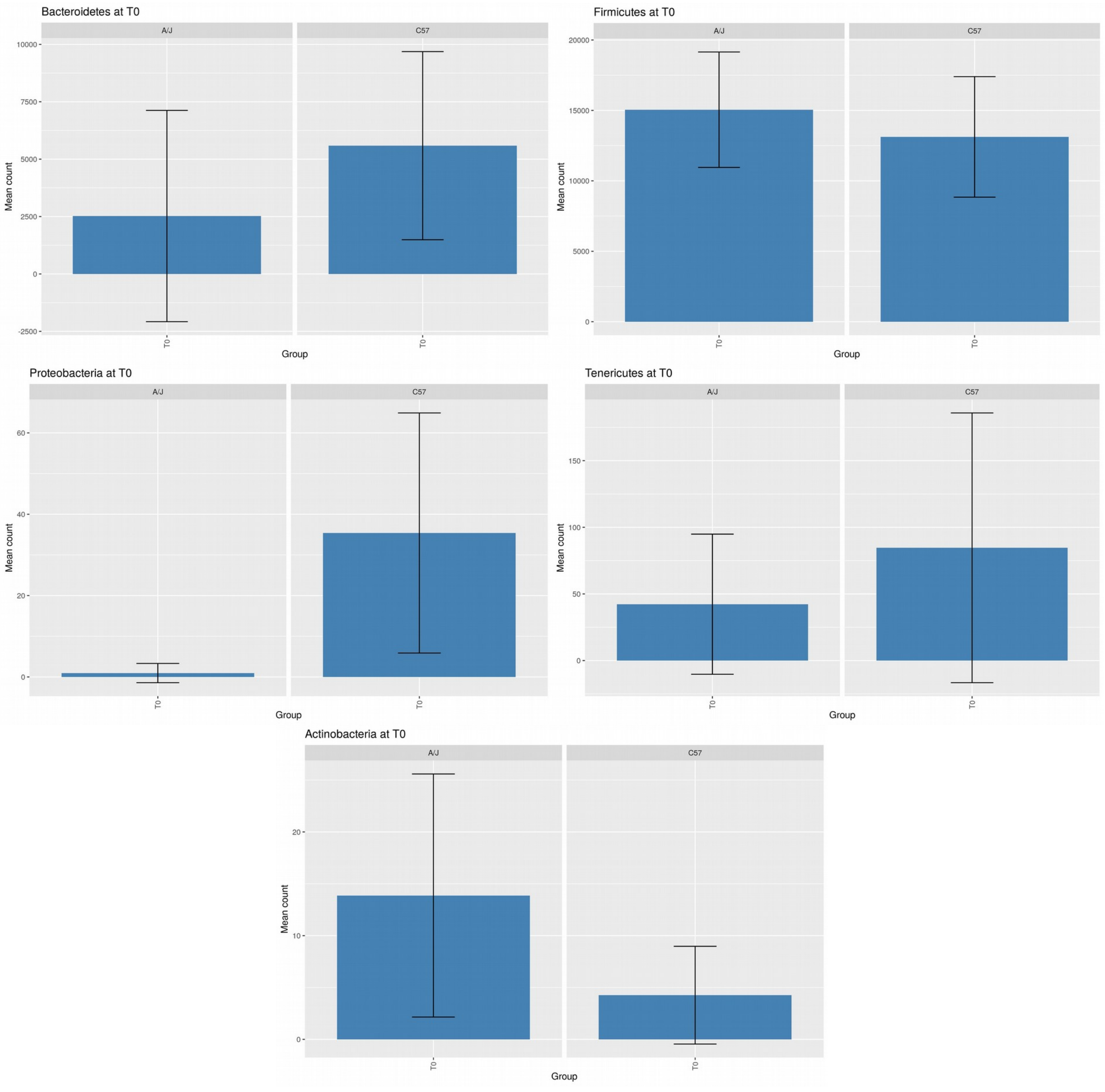

The mean count of different bacteria in A/J and C57 strain before treatment.
