# Supplementary figures and images for: Murine Genetic Background Overcomes Gut Microbiota Changes to Explain Metabolic Response to High-Fat Diet
Source: Nutrients. 2020 Jan 21;12(2):287. doi: 10.3390/nu12020287 (PMC7071469; doi:10.3390/nu12020287)

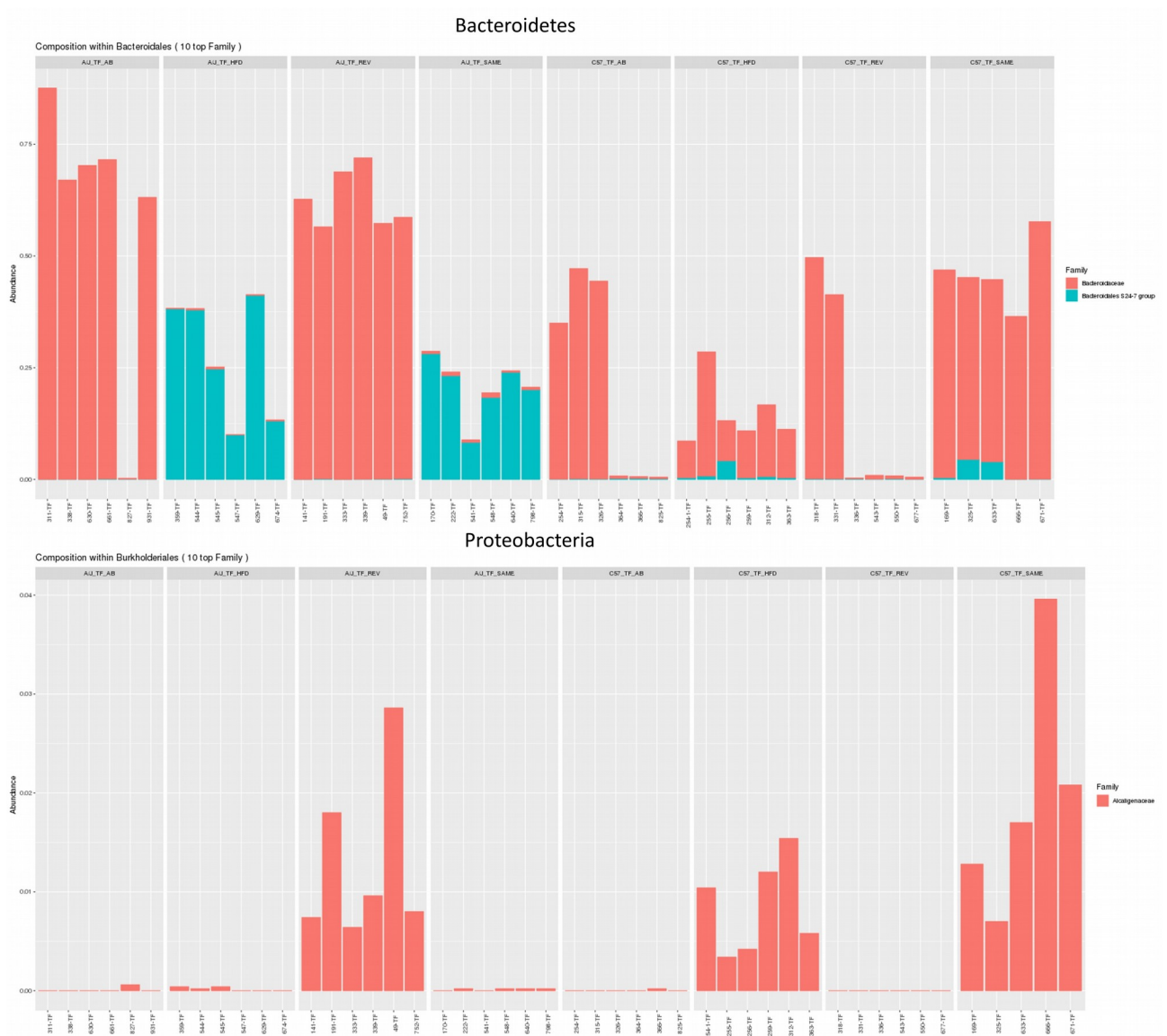

The abundance of some particular bacteria in A/J and C57 mice in different treatment groups.

Supplement: Supplementary file 1 [file nutrients-12-00287-s001.zip › Additional files/Family_Difference_Exchange.pdf]
